# 2′-Alkynyl spin-labelling is a minimally perturbing tool for DNA structural analysis

**DOI:** 10.1093/nar/gkaa086

**Published:** 2020-02-13

**Authors:** Jack S Hardwick, Marius M Haugland, Afaf H El-Sagheer, Denis Ptchelkine, Frank R Beierlein, Andrew N Lane, Tom Brown, Janet E Lovett, Edward A Anderson

**Affiliations:** 1 Chemistry Research Laboratory, University of Oxford, 12 Mansfield Road, Oxford, OX1 3TA, UK; 2 Chemistry Branch, Department of Science and Mathematics, Faculty of Petroleum and Mining Engineering, Suez University, Suez 43721, Egypt; 3 Weatherall Institute of Molecular Medicine, Department of Oncology, University of Oxford, John Radcliffe Hospital, Headley Way, Oxford OX3 9DS, UK; 4 Research Complex at Harwell, Rutherford Appleton Laboratory, Didcot, OX11 0FA, UK; 5 Computer-Chemistry-Center and Interdisciplinary Center for Molecular Materials, Department of Chemistry and Pharmacy, Friedrich-Alexander-Universität Erlangen-Nürnberg, Nägelsbachstrasse 25, 91052 Erlangen, Germany; 6 Center for Environmental and Systems Biochemistry and Department of Toxicology & Cancer Biology, The University of Kentucky, 789 S. Limestone St., Lexington, KY 40536, USA; 7 SUPA School of Physics and Astronomy and BSRC, University of St Andrews, North Haugh, St Andrews KY16 9SS, UK

## Abstract

The determination of distances between specific points in nucleic acids is essential to understanding their behaviour at the molecular level. The ability to measure distances of 2–10 nm is particularly important: deformations arising from protein binding commonly fall within this range, but the reliable measurement of such distances for a conformational ensemble remains a significant challenge. Using several techniques, we show that electron paramagnetic resonance (EPR) spectroscopy of oligonucleotides spin-labelled with triazole-appended nitroxides at the 2′ position offers a robust and minimally perturbing tool for obtaining such measurements. For two nitroxides, we present results from EPR spectroscopy, X-ray crystal structures of B-form spin-labelled DNA duplexes, molecular dynamics simulations and nuclear magnetic resonance spectroscopy. These four methods are mutually supportive, and pinpoint the locations of the spin labels on the duplexes. In doing so, this work establishes 2′-alkynyl nitroxide spin-labelling as a minimally perturbing method for probing DNA conformation.

## INTRODUCTION

**Figure 1.**
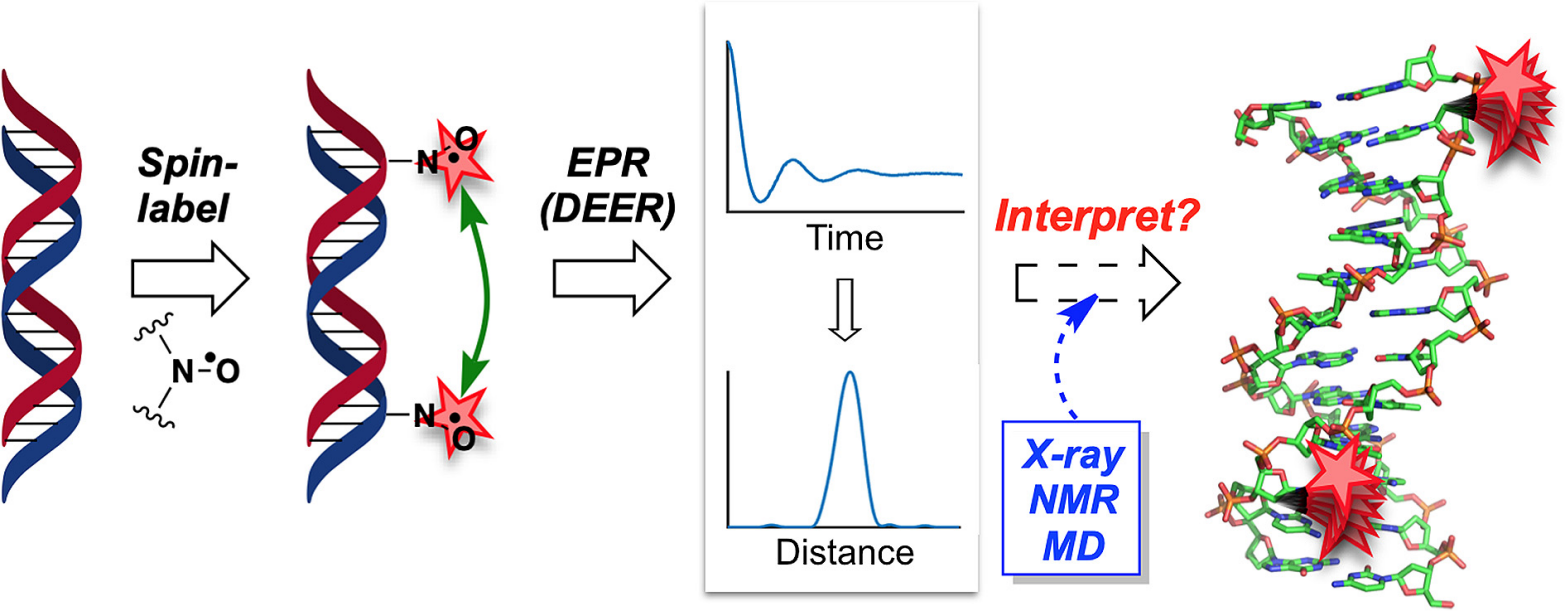
DEER EPR spectroscopy can provide interspin distance distributions. Interpretation in terms of molecular structure can be challenging where the precise location of the spin label is uncertain.

Electron paramagnetic resonance (EPR) spectroscopy can offer unique insight into the structure and dynamics of biomolecules such as DNA by observation of the behaviour of free radicals incorporated into the target of interest (1–3). The pulsed technique double electron–electron resonance (DEER) is particularly useful for the measurement of distances between two paramagnetic centres (typically 1.5–8 nm), termed ‘spin labels’ (Figure 1) (4–10). Provided these labels adopt well-defined positions, DEER can afford distance distributions with sub-nanometre precision, as well as information on their relative orientation (8,11). However, such accuracy depends on the rigidity of the tether between the spin label and the biomolecule, balanced against the inevitable, but ideally minimal, structural perturbation imparted by the chemical modification itself (12). Interpretation of these measurements is challenged by uncertainty in the exact location of the spin label, which hampers the routine use of EPR spectroscopy as an analytical tool in structural biology. This limitation similarly applies to complementary techniques such as Förster resonance energy transfer (13–16), where relatively flexible linkers are commonly used to minimize the uncertainty of the orientation factor (17), but at a cost of sampling a large conformational space, which reduces precision. Improved understanding of the positioning of molecular probes on biomolecules would therefore bring significant benefits in structural biology.

The interpretation of interspin distances in biomolecular EPR typically relies on the use of a complementary technique to correlate spin label position with the EPR-derived most probable distance value (Figure 1). X-ray crystallography would arguably be the most powerful tool for this purpose, as it can provide highly detailed structural information at atomic resolution, in principle without restriction in the size of the biomolecule (18). However, crystallization of spin-labelled nucleic acids is challenging: only one crystal structure of a spin-labelled nucleic acid has been reported, in which a DNA duplex containing two nitroxide-bearing cytidine analogues was crystallized in the A-form (19). Although providing evidence for the non-perturbing nature of this particular spin label, and its positioning in the major groove of A-form duplexes, this solid-state structure very likely differs from the conformation adopted in solution (i.e. a B-form double helix) (20).

Solid-state structures can also be influenced by intermolecular crystal packing effects. Solution-state nuclear magnetic resonance (NMR) spectroscopy is not susceptible to this limitation, and can detect conformational ensembles as well as dynamic structural changes (18). NMR has been used to investigate complexes between proteins and spin-labelled nucleic acids by exploiting the line broadening caused by paramagnetic labels to broadly identify spin label position (21), and by combining short-range distance measurements from NMR with longer-range measurements from EPR (22,23). For nitroxide radicals, reduction to the (diamagnetic) hydroxylamine removes line-broadening effects, and allows NMR-based study of the environment of the (albeit reduced) spin label.

Computational investigations are the most common technique used to aid interpretation of EPR measurements. A detailed picture of a conformational ensemble can be obtained through molecular dynamics (MD) simulations, where consideration of the dynamic behaviour of the spin label, the biomolecular scaffold, and the linker connecting the two, can enable the prediction of one or more conformer classes (7,24–33). In sampling a wide range of conformations, MD simulations are less likely to converge on local (as opposed to global) energy minima than simple energy minimization approaches (34–41), but can be resource-intensive. Dedicated open-source modelling software offers an alternative requiring less expertise (42–48); these methods treat the biomolecular framework as rigid, and consider only the conformational space available by rotation of bonds in the linker and spin label itself. Such algorithms do account for interactions between the label and backbone to different degrees, but they do not consider any label-induced backbone perturbation. Nevertheless, they can afford reasonable models for interpreting EPR distance distributions (49).

In previous work, we described the synthesis of 2′-alkynylnucleotides and their incorporation into DNA duplexes (50), a strategy which permits spin-labelling at any position in an oligonucleotide via click functionalization of a 2′-alkynylnucleotide with azide-functionalized nitroxides (51). The dynamics and separation of the spin labels were studied using continuous wave and DEER experiments, which revealed that these 2′-triazole-linked nitroxides give rise to narrow distance distributions. These showed good agreement with predictions from a basic structural model, but as many different vectors could satisfy the EPR-derived distances, this correlation was likely of limited significance. In the absence of precise knowledge of spin label position and orientation, only qualitative interpretation was possible. This remains a general limitation not only in the bio-EPR field (52), including other methods for 2′-spin-labelling (53,54), but for biomolecule labelling in general.

Here we address this challenge through a synergistic combination of EPR spectroscopy, X-ray crystallography, NMR spectroscopy and MD simulations, which accurately identify spin label location on a DNA duplex. As well as affording the first X-ray crystallographic structures of B-form spin-labelled DNA duplexes, the combination of these four techniques demonstrates the minimally perturbing nature of deoxynucleic acid 2′-spin-labelling. Our results show that EPR spectroscopy with 2′-triazole spin-labelled nucleotides can be used as a tool for accurate and precise distance measurements in DNA, providing a sound basis for the exploration of nucleic acid conformation and dynamics.

## MATERIALS AND METHODS

For all methods, further details and raw data are provided in the Supplementary Material.

### Oligonucleotide synthesis and purification

Oligonucleotides were synthesized with an Applied Biosystems 394 automated DNA/RNA synthesizer, with a standard 1 μmol-scale phosphoramidite cycle of acid-catalysed detritylation, coupling, capping and iodine oxidation. All β-cyanoethyl phosphoramidite monomers were dissolved in anhydrous acetonitrile at a concentration of 0.1 M immediately before use. Synthesis of the 2′-alkynyluridine phosphoramidite and spin-labelling of 2′-alkynyl-modified oligonucleotides (Supplementary Scheme S1 and Table S1) was carried out according to the procedure of Haugland *et al.* (50). An extended coupling time (10 min) was used for the 2′-alkynyluridine phosphoramidite in oligonucleotide synthesis. Oligonucleotides were purified by a RP-HPLC system.

### EPR spectroscopy

The 4-pulse DEER experiments were performed on 200 μM (based on ssDNA) spin-labelled duplexes **5** and **6**, in 150 mM NaCl, 10 mM Na_3_PO_4_, pH 7.4 in D_2_O and 50% glycerol by volume, measured in a Bruker Elexsys E580 high-powered (150 W) Q-band (34 GHz) spectrometer at 50 K. Samples were flash frozen from room temperature (293 K), or from 253 K. A 4-pulse DEER experiment was used to obtain distance distributions (55,56). Further details of the experimental parameters and the results of freezing temperature and concentration are given in the Supplementary Data. DeerAnalysis2016 (57) was used to process the data; Tikhonov Regularisation was used to extract distance distributions using the L-curve method (Supplementary Figures S2, 6 and 7).

### Oligonucleotide crystallization

Duplexes were prepared from the purest HPLC fraction of each oligonucleotide to a concentration of 1 mM duplex in a 10 mM solution of KCl. These stock solutions were used to set up Natrix HT crystallization screens (Hampton Research) in 96-well Greiner plates, using 100 nl DNA solution and 100 nl crystallization reagent solution dispensed by a Microsys instrument (Cartesian Technologies). Plates were sealed and kept at 21 °C in a Formulatrix Storage and Imaging system for 20 days, until suitable crystals had grown.

### X-ray crystallography

The Natrix HT (Hampton Research) screen was used to identify suitable crystallization conditions. Crystals were flash-cooled in liquid nitrogen, and all diffraction data were collected at the Diamond Light Source synchrotron science facility, Harwell, UK, at beamline I04 (100 K, Pilatus 6M hybrid pixel array detector, X-ray wavelengths of 0.979 and 0.916 Å for PDB IDs: 6QJS (duplex **6**) and 6QJR (duplex **7**). Data were indexed and scaled with XDS (58) and AIMLESS (59). Both structures were solved by molecular replacement with Phaser (60) (using PDB IDs: 1S2R and 1D98 as search models for 6QJS and 6QJR); to prevent model bias, all atoms differing between the search model and the structure in question were removed. After an initial stage of refinement with REFMAC5 (61), missing atoms were rebuilt manually with COOT (62), followed by further rounds of refinement. In the crystal of 6QJS twinning was detected according to the L-test. The twin law and twin fraction were determined in AIMLESS. Twin refinement was performed in REFMAC5. For 6QJR, the occupancies of the 5-Me spin label atoms for which no electron density was observed were set to zero during refinement and omitted from the final structure prior to deposition. Quantitative structural analysis was performed with 3DNA (63) and Curves+ (64); RMSDs were calculated in PyMOL, and interspin distances were determined using UCSF Chimera (65).

### Molecular dynamics simulations

All-atom, explicit solvent MD simulations of duplexes **5** and **6** were performed with Amber 16 (66) and Amber 18 (67), using A- and B-DNA conformations of each duplex as starting structures. Parameter derivation, system set-up and the simulations were performed following a protocol established previously (14).

### NMR spectroscopy

Three 14-mer oligodeoxynucleotides containing the Mbp1 binding site (68) were synthesized and purified as described above: 1. dCAATGACGCGTAAG; 2. dCT^5NO^UACGCGTCATTG; 3. dCT^6NO^UACGCGTCATTG. Strand 1 is complementary to stands 2 and 3, which were modified at position 3 by substitution of a 2′-spin-labelled uridine residue (denoted ^5NO^U or ^6NO^U) for thymidine. Lyophilized oligonucleotides were dissolved in 20 mM sodium phosphate, 80 mM KCl, pH 7 in 99.9% D_2_O + 23 μM DSS (sodium 2,2-dimethyl-2-silapentane-5-sulfonate), and duplexes **9** and **10** were formed by mixing equimolar amounts of 1 with 2 or 3, followed by slow annealing from 60 °C. All NMR spectra were recorded at 14.1 T on an Agilent DD2 spectrometer using a 3 mm inverse triple resonance HCN cold probe. After recording spectra of the nitroxide-containing duplexes, 5 μl aliquots of 1 M sodium dithionite dissolved in the same buffer were added to reduce the nitroxide to the hydroxylamine diamagnetic state, and NMR spectra were recorded again on the reduced (hydroxylamine) form of the duplexes (**9^NOH^** and **10^NOH^**).

## RESULTS

### DNA synthesis and EPR spectroscopy

**Figure 2.**
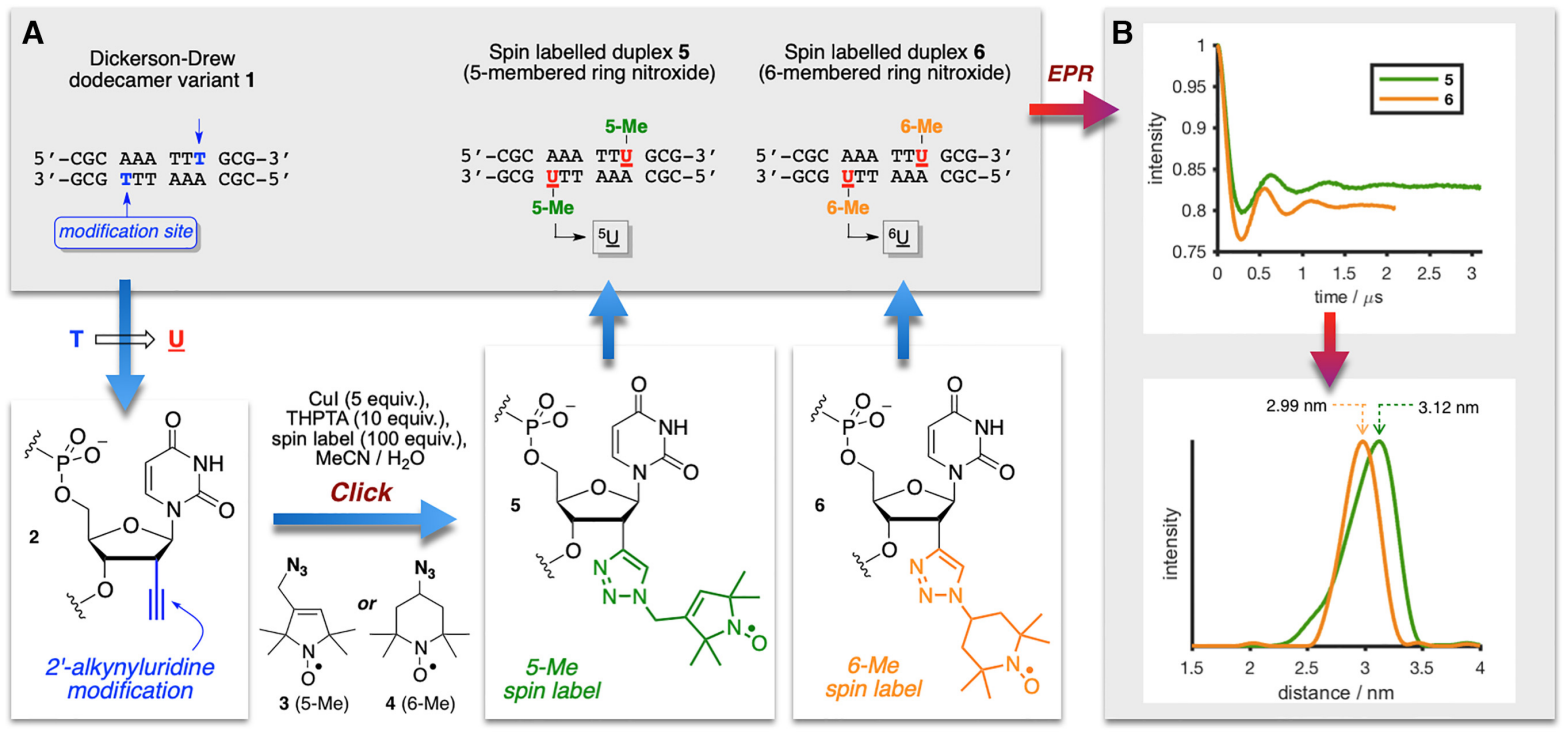
Synthesis of spin-labelled Dickerson–Drew dodecamer variants and DEER experiments. (**A**) Synthesis of spin-labelled DNA duplexes by modification of the 9-position in duplex **1** as a 2′-alkynyluridine (**2**), and incorporation of nitroxide radicals **3** (5-Me) and **4** (6-Me) into the spin-labelled duplexes **5** and **6**. (**B**) DEER-derived interspin distance distributions for duplexes **5** and **6** (200 μM ssDNA); the most probable interspin distances are 3.12 nm (**5**) and 2.99 nm (**6**). Data were analysed using DeerAnalysis2016 (57). THPTA = tris(3-hydroxypropyltriazolylmethyl)amine.

To compare distance distributions obtained from DEER experiments with those determined or predicted by other techniques, we sought a DNA sequence amenable to crystallization in the B-form (the predominant form of DNA in solution), and suitable for analysis by EPR spectroscopy. A variant of the Dickerson–Drew dodecamer was selected which, unmodified, has been crystallized in the B-form (**1**, Figure 2) (69). Sequence **2**, containing a 2′-alkynyluridine (U) in place of thymidine at the 9-position, was synthesized according to our previous studies (50). Copper-catalysed azide-alkyne cycloaddition (70) with a tetramethylpyrrolinoxyl radical (‘5-Me’, **3**) or a tetramethylpiperidinoxyl radical (‘6-Me’, **4**) afforded duplexes **5** (containing two ^5^U modifications) and **6** (two ^6^U modifications). These nitroxides were selected on the basis of our previous work on different duplexes, where well-defined, but distinct conformations were observed for each nitroxide (50).

The double spin-labelled duplexes **5** and **6** were studied by EPR spectroscopy (Supplementary Figures S1 and 2), using DEER experiments to determine distance distributions between the two radicals (Figure 2B). We found that the modulation depth of the DEER time trace, which is related to the equilibrium between single-stranded and duplex DNA in the (frozen) solution, depended on the temperature of the sample before rapid (shock) freezing in liquid nitrogen. Specifically, samples that were frozen from a pre-cooled state (253 K) afforded a higher concentration of duplex (deeper modulation) than those frozen from room temperature; the DEER time traces and distance distributions were essentially identical irrespective of the method of cooling (See Supplementary Figures S3–5). This is consistent with previous observations on the influence of the ‘shock-freezing’ method on spin label distance distribution (71), and the expected equilibrium distribution in solution at 253 K under the salt and strand concentrations used.

From the analysed DEER data, both spin labels were found to exhibit narrow distributions (fwhh 0.48 and 0.37 nm, respectively for **5** and **6**). Duplex **6** displayed a near symmetric distribution with a most probable interspin distance of 2.99 nm, suggesting a distribution of very similar conformations of the spin label; the distribution for duplex **5** was less symmetric, implying a broader conformational ensemble, with a most probable interspin separation of 3.12 nm. The distributions show that the spin labels occupy well-defined locations on the duplexes.

### X-ray crystallography

**Figure 3.**
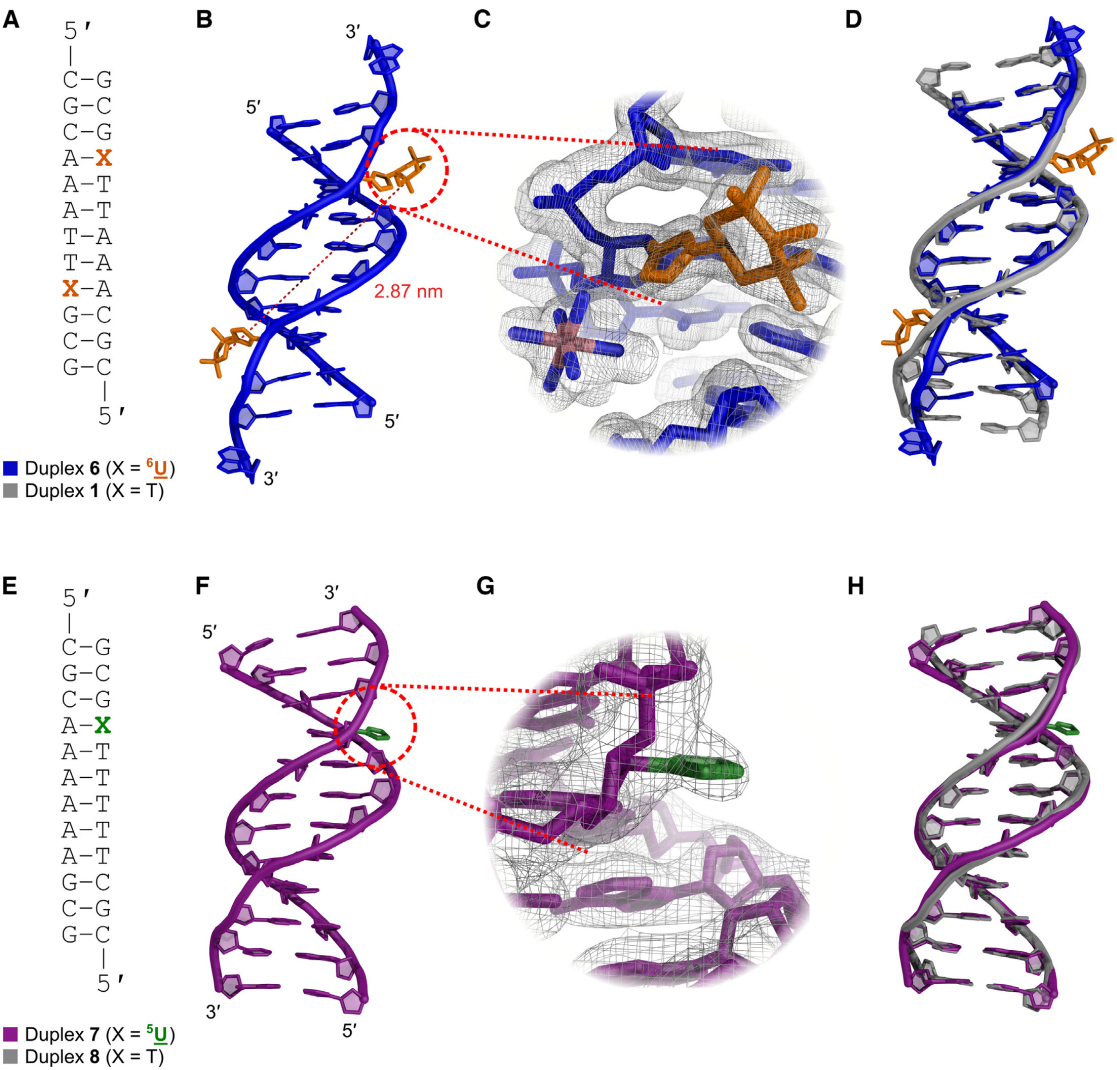
B-form crystal structures of spin-labelled DNA duplexes. (**A**) Sequence of duplex **6** (X = ^6^U) and its unmodified counterpart duplex **1** (X = T). (**B**) Crystal structure of **6** (PDB ID: 6QJS). The spin labels (orange) occupy the minor groove and are separated by 2.87 nm. (**C**) A section of the 2*F*_o_-*F*_c_ electron-density map contoured at 1.0 σ, showing one of the 6-Me spin labels. (**D**) Overlay of **6** (blue) and unmodified duplex **1** (grey, PDB ID: 1S2R). (**E**) Sequence of duplex **7** (X = ^5^U) and its unmodified counterpart duplex **8** (X = T). (**F**) Crystal structure of **7** (PDB ID: 6QJR). The electron density of the 5-Me spin label is not well-defined; only that of the triazole (green) can be observed (**G**). (**H**) Overlay of **7** (purple) and **8** (grey, PDB ID: 1D98).

To facilitate a detailed structural interpretation of the DEER distance measurements, the spin-labelled duplex was investigated using complementary techniques. We first turned to X-ray crystallography, where we obtained a 1.8-Å structure of duplex **6** (PDB ID: 6QJS, Supplementary Tables S2–6), which crystallized in the *P*3_2_21 space group with a single duplex in the asymmetric unit (Figure 3A). Notably, **6** adopts the B-form; prior to this work, the only reported crystal structure of spin-labelled DNA is in the A-form (19). The electron density of the 6-Me spin labels is well defined, with both modifications occupying the minor groove. The separation of the spin labels (measured from the centre of each N–O bond) is 2.87 nm, which is in good agreement with the most probable distance determined by EPR spectroscopy (2.99 nm). The electron density of all other residues is also well defined, except for the 5′-terminal 2′-deoxycytidines, whose base-pairing is disrupted by crystal-packing interactions with cobalt(III) hexammine ions and neighbouring duplexes in the crystal (see Supplementary Figure S8). Such crystal contacts are commonly observed in DNA structures containing both terminal CpG steps and cobalt ions (72–79) and are thus not attributable to the inclusion of the spin labels, which are separated from the terminal residues by two intact base pairs.

The specific conformations adopted by the triazole linker and spin label merit consideration: as shown in Figure 3C, the deoxyribose, triazole and piperidine rings adopt conformations that minimize torsional strain, and steric effects. The triazole ring is oriented with a nitrogen atom positioned under the deoxyribose ring, which places the triazole carbon atoms and sidechain in a less hindered environment. Similarly, the piperidine C–C bonds at the linking carbon atom minimize steric interactions by adopting a gauche conformation relative to the N1–N2 bond of the triazole. This may suggest that despite having two rotatable bonds between the deoxyribose and piperidine, local conformational effects greatly restrict the conformations available to the system, and in particular the triazole.

Efforts to obtain well-diffracting crystals of the ^5^U analogue duplex **5** were unsuccessful; however, we could determine the structure of the related single spin-labelled duplex **7** (Figure 3E) at 2.9-Å resolution, which also crystallized in the B form, in the *P*2_1_2_1_2_1_ space group (Figure 3F and Supplementary Tables S2–6, PDB ID: 6QJR). The electron density of all residues is well-defined, but only a small amount of density is seen for the 5-Me spin label, corresponding to the triazole (see also Supplementary Figure S9). The lack of density is likely due to dynamic disorder associated with the methylene linker. Although the pyrroline ring is not resolved, the triazole is again observed to adopt a conformation with its N3 atom situated under the deoxyribose ring.

Superposition of the spin-labelled duplexes **6** and **7** with their unmodified counterparts (PDB IDs: 1S2R and 1D98, respectively) showed only minor perturbation of the global structure, despite significant differences in crystal packing, as assessed by their all-atom RMSDs (1.08 Å, 402 atoms and 0.70 Å, 403 atoms respectively, omitting the terminal bases to minimize crystal-packing effects, Figure 3D/H). These values fall well within the range of RMSDs between DNA duplexes of identical sequence that have been crystallized under different conditions (20). Most notable is a difference in groove widths for part of duplexes **1** and **6**, where the AAATTT tract in the crystal structure of **1** has an unusually narrow minor groove (Figure 3D) (69). This narrowing is not seen in the structure of **6**, either due to the presence of the spin label, or to differences in crystal packing (see Supplementary Figure S10). By contrast, superposition of the X-ray structures of duplexes **7** and **8** (Figure 3H) shows that their overall geometry is highly conserved.

Although duplexes **6** and **7** exhibit overall B-form geometry, some local structural parameters varied at the modified uridines. Most notably, the puckering and glycosyl torsion angles of these residues are A-form-like: the pseudorotation phase angles *P* of the ^6^U sugars are 49° and 36° (C4′-*exo* and C3′-*endo*), and 45° for the ^5^U sugar (C4′-*exo*), whilst the glycosyl torsion angles are –141° and –146° for the ^6^Us, and –146° for ^5^U (see Supplementary Tables S7–9). The similarity of the spin-labelled uridine phase angles, despite differences in their crystalline environments, suggests that the observed pucker represents a major conformer rather than a crystal-packing effect.

Deviation from B-form geometry at these sites is not unexpected, as even single atom 2′-substituents are known to substantially alter sugar puckering (80). Although only two other B-DNA structures in the PDB contain 2′-modified ribonucleotides, the modified sugars in those duplexes also adopt an A-DNA-like conformation (C3′-*endo*, see Supplementary Figure S11) (81,82). Importantly, the perturbations in duplexes **6** and **7** are localized at the modified nucleotides and do not propagate along the duplex (Supplementary Tables S8 and 9), which would have significant implications for the EPR distance distributions. Overall, these crystallographic studies demonstrate the compatibility of the 2′-spin-labelling strategy with B-form DNA. Local deviations at the modification sites do not significantly perturb the global structure of the double helices implying, in the solid state at least, that oligonucleotides labelled in this manner are structurally representative of unlabelled target sequences.

### Molecular dynamics simulations

To understand the spin label distance distributions obtained from EPR spectroscopy, we undertook *in silico* investigations of the dynamic solution-phase behaviour of the double spin-labelled duplexes **5** and **6**, which provided an opportunity to identify possible modes of spin label localization (see the Supplementary Data for computational details). MD simulations (14,83) of duplex **6** revealed two major duplex conformations, featuring distinct orientations of the 6-Me spin labels. One of these conformations arose from a 1000 ns simulation starting from an A-form duplex (sugars C3′-*endo*) which converted to the B-form within 3 ns, the other from simulating a B-form duplex over 1000 ns (sugars C2′-*endo*) (84).

**Figure 4.**
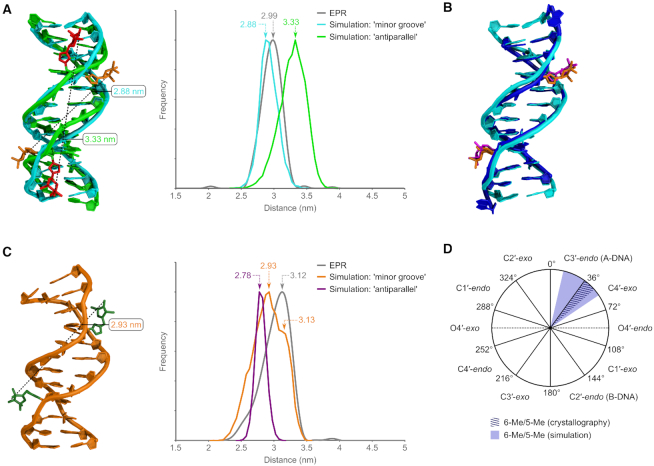
MD simulations of duplexes **5** and **6**. (**A**) Two conformations and distance distributions are observed for **6**, with the spin label oriented in the minor groove (cyan), and antiparallel (green). The EPR distance distribution is shown in grey. (**B**) Overlay of a snapshot from the minor groove cluster and the X-ray structure of **6**. (**C**) Simulations of **5** reveal a conformation with the 5-Me spin label in the minor groove (orange). Distance distributions for a simulated antiparallel conformation (purple) and the EPR data (grey) are also shown. (**D**) Comparison of phase angles between simulation and X-ray crystallography show the modified nucleotides adopt similar A-form like conformations (see Supplementary Tables S10 and 11).

In the former conformation, the label associates with the minor groove, and exhibits a distance distribution for all conformations (between 200.5–1000.5 ns) that correlates very well with the DEER-derived distance distribution (see Supplementary Figure S13 for a picture of the conformational ensemble). A simulation snapshot was selected from the most populated minor groove cluster (which comprises 37% of the snapshots of the corresponding simulation) in which the interspin distance is closest to the maximum of the calculated distance distribution (Figure 4A and Supplementary Figure S12, cyan, most probable interspin distance 2.88 nm). In the second conformation, the labels orient antiparallel with respect to the helix, with the nitroxides solvent-exposed but close to the major groove (Figure 4A, green, most probable distance 3.33 nm). As depicted in the overlay in Figure 4B, the minor groove conformation is consistent with the duplex structure and nitroxide locations observed by X-ray crystallography (interspin distance 2.87 nm), with an RMSD of 1.08 Å (402 atoms, excluding the terminal nucleotides and spin labels); the simulated and experimental (EPR, 2.99 nm) interspin distances show similar agreement. The antiparallel conformation predicted from the second simulation does not appear to contribute significantly in the ‘real’ system. These two conformations cannot interconvert in the MD simulations where complete duplex formation is maintained throughout.

Similar minor groove and antiparallel conformations were found for duplex **5** (Figure 4C; Supplementary Figures S14 and 15). However, the 5-Me spin label was significantly more conformationally flexible than the 6-Me label, and several variants of the minor and major groove conformations were observed where the pyrrolinoxyl ring points away from the duplex (in the case of the minor groove conformation, reminiscent of electron density observations from the X-ray studies on duplex **7**), or associates with the major groove (in the case of the antiparallel conformation). The experimental maximum is now seen as a significant shoulder in the simulation (3.13 nm, see the Supplementary Figure S15 for a picture of the conformational ensemble), indicating a different relative weighting of the individual conformers between simulation and experiment. Nonetheless, these simulations support minor groove localization of the nitroxide, as no conformers with antiparallel nitroxide orientation were observed with interspin distances that agree with the experimental measurement.

Analysis of the phase angles (85) of the ^6^U nucleotides in the snapshot of duplex **6** (Figure 4A, cyan; see Supplementary Figures S18 and 19 for full ensemble data) revealed puckers of C4′-*exo* (56°) and C3′-*endo* (13°), conformations that were also observed crystallographically (49° and 36°, see Figure 4D and Supplementary Table S10). The other nucleotides adopt B-form geometry (mean phase angles across the ensemble 148° ± °6, C2′-*endo*). As with the X-ray structures, the triazole ring primarily occupies the least sterically hindered environment, with the triazole N3 atom positioned under the ribose ring; the C–C bond linked to the spin label again adopts a gauche conformation (Supplementary Figures S22 and 23).

The sugars of the ^5^U nucleotides in the minor groove conformation of duplex **5** show similar values; in the snapshot in Figure 4C, C3′-*endo* conformations with angles of 19° and 29° are observed, with other nucleotides adopting the B-form (see Supplementary Figures S20 and 21 for full ensemble data; mean phase angles across the ensemble 147 ± 6°, C2′-*endo*). These angles are similar to that observed for the ^5^U nucleotide in the crystal structure of the singly modified duplex **7** (C4′-*exo*, 45°, Figure 4D and Supplementary Figure S11).

Analysis of the glycosyl torsion angles also revealed excellent agreement between the MD simulations and the X-ray structures. For the minor groove conformation of duplex **6** in Figure 4A, angles of –145° and –155° were obtained for the two ^6^U nucleotides, compared to values of –141° and –146° in its X-ray structure (Supplementary Table S11). Angles of –139° and –144° for duplex **5** also match very well with the X-ray value of –146° in duplex **7**.Overall, given that X-ray crystallography provides only a snapshot of a conformational ensemble, the finding that similar conformations are possible and indeed favoured *in silico* strongly supports the proposed minor groove assignment of spin label localization.

### NMR spectroscopy

**Figure 5.**
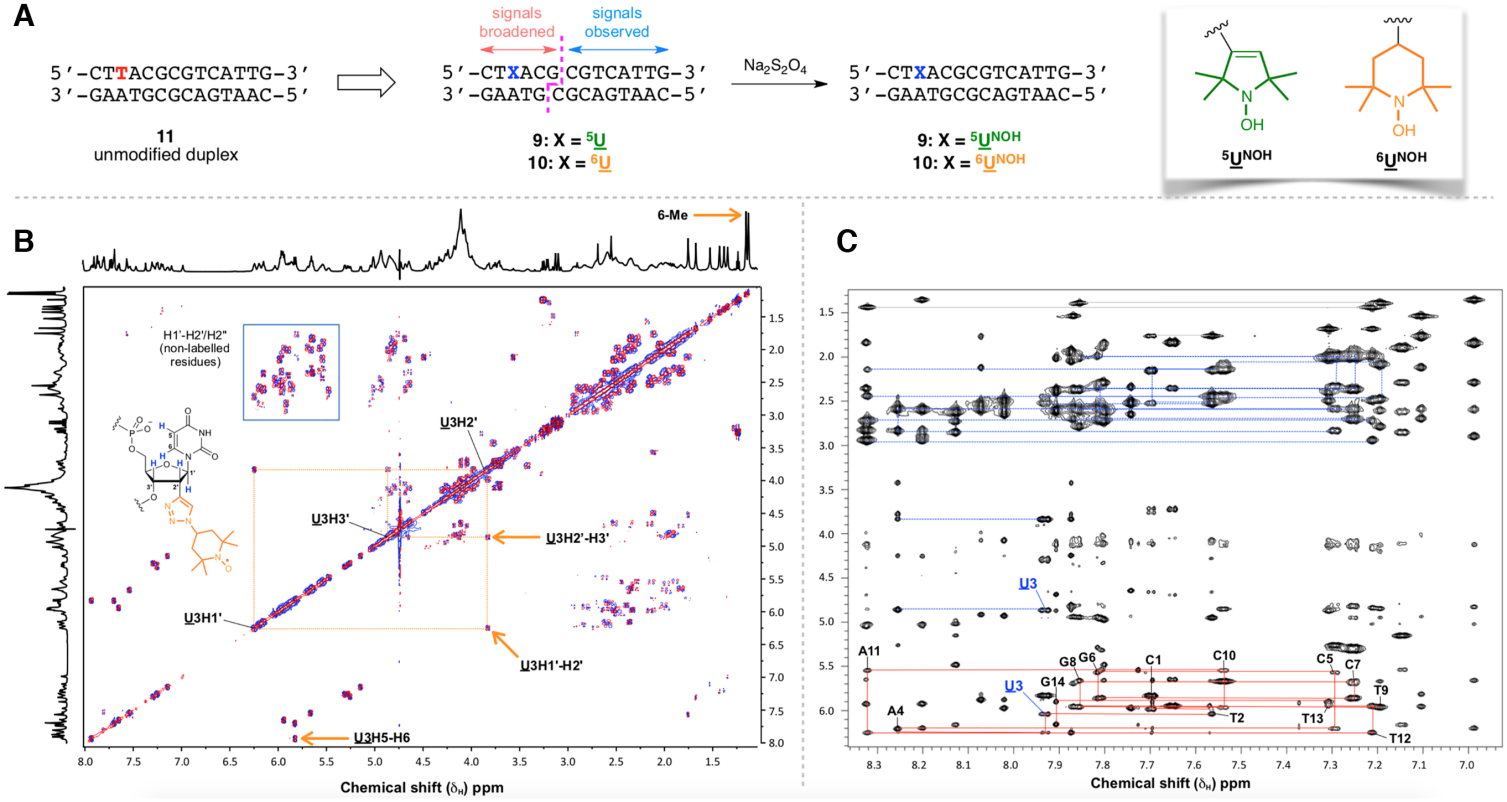
NMR spectroscopic analysis. (**A**) Unmodified duplex **11**, and the single spin-labelled duplexes **9** and **10**. The pink dashed line divides the nucleotides into those whose resonances are broadened beyond detection by the nitroxide, and those that can be detected. Reduction of the nitroxide to the hydroxylamine gives duplexes **9^NOH^** and **10^NOH^**. (**B**) ^1^H–^1^H DQF-COSY spectrum for **10^NOH^** at 30 °C. The key chemical shift changes arising from the 2′-triazole modification are indicated. (**C**) The 200 ms ^1^H–^1^H NOESY spectrum for **10^NOH^** at 30 °C. Assignments of the modified strand are shown (red). Dashed lines (blue) show the sequential H2′/H2′ to H8/6 connectivities. The relative base-H1′ and H2′ intensities are consistent with an overall B conformation.

NMR spectroscopy offers a different means to explore the consequences of duplex spin-labelling on the conformational ensemble. As nitroxides cause extreme line broadening over distances up to ∼1.2 nm (∼4 bp in B-DNA), double spin-labelled duplexes **5** and **6** would show few NMR signals in the paramagnetic state. We therefore instead examined the non-self-complementary duplexes **9** and **10** containing single spin labels (^5^U3 and ^6^U3 respectively, Figure 5A and Supplementary Figure S24), whose native structure **11** has previously been determined by NMR spectroscopy (68). In the diamagnetic state (with the nitroxide radicals reduced to hydroxylamines, duplexes **9^NOH^** and **10^NOH^**), resonances were assigned for all nucleotides (see Supplementary Tables S12–14). The chemical shift differences between these diamagnetic duplexes are insignificant, implying that the overall conformations at the sites of modification are very similar (Supplementary Figure S25). Further, the chemical shift differences between the two non-reduced duplexes and unmodified duplex **11** are insignificant between G6:C23–G14:C15, whereas there are more significant conformational changes at the modified uridines, and their nearest neighbours, on both strands (Supplementary Figure S26).

The NOESY peak intensities and coupling constants of the sugar protons (obtained from 1D, DQF-COSY and NOESY spectra, Figure 5B/C) confirmed no significant differences in conformation for the G6:C23–G14:C15 segment, indicating that any conformational changes due to the modified nucleotides are local. dT→dU modification alone has little effect on the conformation of DNA duplexes in the solution state (86,87), although measurable changes in chemical shifts are expected at the site of modification and its nearest neighbours. However, 2′-spin-labelling affects not only the chemical shifts of the ribose protons of the modified U nucleotides, but also the sugar puckering. In native duplex **11**, all nucleotides are predominantly C2′-*endo* with a small admixture of C3′-*endo* (^3^*J*_1′2′_ ∼10 Hz and ^3^*J*_2′3′_ ∼6 Hz) (68). This is also the case for all nucleotides except U3 in the modified duplexes (see DQF-COSY Figure 5B; Supplementary Figures S27, 28 and Table S15): the values of ^3^*J*_1′2′_ and ^3^*J*_2′3′_ were 7.0 and 6.0 Hz for ^5^U3 (in duplex **9^NOH^**), and 6.5 and 6.0 Hz for ^6^U3 (in **10^NOH^**).

These values differ significantly from those expected for C2′-*endo* or pure C3′-*endo*, but can be rationalized by a similar proportion of C2′-*endo* and O4′-*endo* states, reflecting the tendency of these sugars towards A-form like conformations. The ^5/6^U glycosyl torsion angles were estimated to be –140 to –150°, comparing well with those determined by crystallography and MD simulations. The chemical shifts of the reduced duplexes are very similar, including at the sites of modification, as are the cross-peak intensities in DQF-COSY and short mixing time (50 ms) NOESY spectra, showing minimal effect of the labels on the overall duplex conformations. The signals corresponding to the methyl groups of the reduced spin labels were sharp, and showed few nOes to the DNA, which is consistent with their occupying the minor groove.

In the paramagnetic states, no resonances were observed for either duplex for the first six bases in the U3-containing strand (C1–G6), or for the final five on the complementary strand (C24–G28, see Supplementary Tables S16, 17 and Figure S29). The chemical shifts of the non-broadened nucleotides C7–G14 and C15–C23 were negligibly different between the oxidized and reduced forms, and unlabelled duplex **11**. The scalar coupling and nOe cross-peak patterns were also essentially identical, implying no differences in conformation due to the paramagnetic state of the label. Overall, NMR analysis shows that 2′-spin-labelling exerts only a local influence on duplex structure.

## DISCUSSION

Interspin distance measurements have the potential to afford detailed information on duplex structure, provided that accurate models can be constructed that enable confident interpretation of EPR results. Analysis of the positioning of 6-Me and 5-Me nitroxides on single and double spin-labelled duplexes showed a remarkable degree of consistency between the results of X-ray crystallography (for **6**), MD simulations and NMR spectroscopy, with the first two of these methods revealing conformations that correlate well with the interspin distance distributions measured by EPR spectroscopy (e.g. duplex **6**, most probable distances 2.87–2.99 nm).

The corresponding analysis for duplexes labelled with 5-Me nitroxides is less conclusive, due to the population of several conformations of the 5-Me spin label by free rotation around the methylene linker. Nevertheless, the X-ray structures of both the double 6-Me-labelled duplex **6** and single 5-Me-labelled duplex **7** revealed that the triazole-nitroxide occupies the minor groove of the duplexes. Although the spin labels imparted some local A-DNA-like character at the modification sites, they caused minimal perturbation to global structure, as judged by RMSD values. These observations were reinforced by MD simulations, where antiparallel and minor groove spin label conformations were found within B-form duplexes. For duplex **6**, the simulated minor groove conformation is consistent with the experimental DEER data; the alternative antiparallel conformation is proposed to be a local energy minimum when compared to the more stable minor groove positioning. The calculated sugar puckers and glycosyl torsion angles for the minor groove conformation closely resemble those observed in the solid state. Notably, the agreement between the MD simulations and experimental data is better than that expected using simpler modelling methods (albeit applied to different labels) (42–43,49).

NMR spectroscopic investigations of two single spin-labelled duplexes in the paramagnetic (nitroxide) and diamagnetic (hydroxylamine) forms support adoption of the B-form in solution, with conformations very similar to an unmodified duplex. The spin label likely occupies the minor groove, and once again an A-form like conformation was observed at the modified sugar. Only minor conformational changes were seen at the nucleotides neighbouring the ^5/6^U modification. Our results show that the more rigid linker / spin label **4** (6-Me) imparts similar perturbation of the DNA structure to **3** (5-Me); in the absence of other considerations such as reduction stability, **4** would thus be the label of choice for 2′-alkynyl spin-labelling, given its narrower and near-symmetric EPR distance distributions. Taken together, the three techniques used in this study each establish minor groove positioning of the 2′-triazolyl nitroxide spin labels, thus allowing confident interpretation of associated EPR data and enabling the reliable use of this spin-labelling strategy to probe nucleic acid structure.

These observations lead to a model that explains spin label conformation. The introduction of a 2′-substituent onto the deoxyribose ring leads to a more A-form like pucker. The triazole ring and carbocyclic spin label then occupy a conformationally restricted environment on the sugar that minimizes torsional and steric strain; finally, further conformational restrictions imparted by the constrained environment of the minor groove result in a well-defined location of the spin label. Indeed, it is possible that a significant proportion of the EPR measured distance distribution derives from a range of duplex conformations, as much as from label and linker flexibility itself.

## CONCLUSION

Using experimental and computational methods to support EPR spectroscopic observations, we demonstrate that spin-labelling of DNA via 2′-triazole-linked nitroxides is minimally perturbing and affords DEER data with narrow (sub-nanometre) distance distributions due to well-defined minor groove nitroxide conformations. This multi-faceted approach offers unprecedented insight into spin label localization, enabling more confident interpretation of DEER-derived distance distributions of 2′-triazolyl-mounted spin labels. Overall, this work provides the foundation for further applications of 2′-alkynyl spin-labelling in the study of nucleic acid structure, dynamics and interactions with other biomolecules.

## DATA AVAILABILITY

EPR data is deposited at DOI: 10.17630/c4d64b89-d19b-4f9e-8409-762b430d72f5. Atomic coordinates and structure factors for the reported crystal structures have been deposited with the Protein Data Bank under accession numbers 6QJS and 6QJR. NMR data have been deposited with the Biological Magnetic Resonance Data Bank under accession number 27958.

## Supplementary Material

gkaa086_Supplemental_File
