# Supplementary material for: 2′-Alkynyl spin-labelling is a minimally perturbing tool for DNA structural analysis
Source: Nucleic Acids Res. 2020 Feb 13;48(6):2830–40. doi: 10.1093/nar/gkaa086 (PMC7102949; doi:10.1093/nar/gkaa086)
Supplement: gkaa086_Supplemental_File [file gkaa086_supplemental_file.pdf]

2'-Alkynyl spin-labelling is a minimally perturbing tool for DNA structural analysis

Jack S. Hardwick, Marius M. Haugland, Afaf El-Sagheer, Denis Ptchelkine,* Frank R. Beierlein,* Andrew N. Lane,* Tom Brown,* Janet E. Lovett,* and Edward A. Anderson*

Supplementary Material

Contents

List of Supplementary Figures, Tables and Schemes S2

1. Oligonucleotide synthesis and purification S5

2. EPR spectroscopy S7

3. X-ray crystallography S14

4. Molecular dynamics simulations S22

5. NMR spectroscopy S38

6. References S50

Principal corresponding author: edward.anderson@chem.ox.ac.uk;

Additional correspondence:

DNA synthesis: tom.brown@chem.ox.ac.uk

EPR: jel20@st-andrews.ac.uk

MD simulations: frank.beierlein@fau.de

X-ray crystallography: denis.ptchelkine@rc-harwell.ac.uk

NMR: Andrew.lane@uky.edu

Supplementary Schemes

Scheme	Title	Page
1	Synthesis of the 2'-alkynyluridine phosphoramidite used in this work	S5

Supplementary Figures

Figure	Title	Page
1	Room temperature CW EPR spectra of the spin-labelled DDD in buffer/glycerol	S8
2	DEER time trace and distance distributions	S9
3	DEER time traces for spin-labelled duplexes **5** and **6** at different concentrations and freezing methods	S10
4	Modulation depths from Figure S3 used to estimate the percentage duplex formation	S11
5	DEER time traces for spin-labelled duplexes for samples frozen from 293 K or 253 K (“pre-cooled”)	S12
6	Duplex **5.** Validation of distance certainty using DeerAnalysis2016	S13
7	Duplex **6.** Validation of distance certainty using DeerAnalysis2016	S13
8	The terminal base pairs of duplex **6** are disrupted by crystal-packing interactions with cobalt(III) hexammine ions and symmetry mates	S19
9	$2F_o$-F_c electron density maps of spin-labelled DNA duplexes, contoured at 1.0 σ	S19
10	Groove dimensions of spin-labelled DNA duplexes and their unmodified counterparts	S20
11	Pseudorotation wheel, showing the ranges of phase angles of the 2′-substituted nucleotides from all eleven B-DNA crystal structures containing such modifications deposited in the PDB at the time of writing	S21
12	Simulation of system **6**, starting from B-DNA	S25
13	Simulation of system **6**, starting from A-DNA	S26
14	Simulation of system **5**, starting from B-DNA	S27
15	Simulation of system **5**, starting from A-DNA	S28
16	Three independent runs of system **6**, minor groove conformation	S29
17	Three independent runs of system **5**, minor groove conformation	S30
18	Mean value of the phase angles describing the sugar pucker of all residues vs. time. Simulation of **6** with the spin labels in the minor groove	S32
19	Phase angles of each DNA residue (mean values over 200.5-1000.5 ns) versus sequence. Simulation of **6**, minor groove spin label conformation	S32
20	Mean value of the phase angles describing the sugar pucker of all residues vs. time. Simulation of **5** with the spin labels in the minor groove	S33
21	Phase angles of each DNA residue (mean values over 200.5-1000.5 ns)	S33

	versus sequence. Simulation of **5**, minor groove spin label conformation	
22	Dihedral C1‘-C2‘-C11-N5 in spin label 6-Me in $9^{6}\underline{U}$ and $21^{6}\underline{U}$, and dihedral N4-N30-C13-C14 in $9^{6}\underline{U}$ and $21^{6}\underline{U}$. Simulation of duplex **6**, minor groove spin label conformation	S36
23	Dihedral C1‘-C2‘-C11-N5 in spin label 5-Me in $9^{5}\underline{U}$ and $21^{5}\underline{U}$, dihedral N4-N30-C13-C17 in $9^{5}\underline{U}$ and $21^{5}\underline{U}$, and dihedral N30-C13-C17-C16 in $9^{5}\underline{U}$ and $21^{5}\underline{U}$. Simulation of duplex **5**, minor groove spin label conformation	S37
24	Synthesis of duplexes **9**,**10**, **9**^{NOH} and **10**^{NOH}	S38
25	Chemical shift difference for **10**^{NOH} **–** **9**^{NOH}	S45
26	Chemical shift difference for **10**^{NOH}**–11** and **9**^{NOH}**–11**	S46
27	Double Quantum filtered COSY of the reduced 6Me duplex **10**^{NOH}	S47
28	^{1}H–^{1}H NOESY spectrum of duplex **10**^{NOH}	S48
29	Overlay of ^{1}H NMR spectra of duplex **10** in the oxidized (**10,** red) and reduced (**10**^{NOH}, grey) states	S49

Supplementary Tables

Table	Title	Page
1	Mass spec and UV melting data for modified oligonucleotides	S6
2	Sequences of oligonucleotides used for crystallization	S15
3	Conditions for oligonucleotide crystallization	S15
4	X-ray data collection statistics	S15
5	Twinning statistics for duplex **6** (6QJS)	S16
6	Refinement statistics for duplexes **6** and **7** (6QJS and 6QJR)	S16
7	Phase angles and glycosyl torsion angles for duplexes **6** and **7**	S16
8	Sugar puckers and pseudorotation phase angles of crystal structures PDB 6QJS (containing 6-Me-labelled uridine), PDB 6QJR (containing 5-Me-labelled uridine), and those of their unmodified counterparts PDBs 1S2R and 1D98, respectively	S17
9	χ torsion angles of crystal structures PDB 6QJS (containing 6-Me-labelled uridine), PDB 6QJR (containing 5-Me-labelled uridine), and those of their unmodified counterparts PDBs 1S2R and 1D98, respectively	S18
10	Phase angles for modified nucleotides in the snapshot in Fig. 4 in the main text, and mean / standard deviations over 200.5–1000.5 ns	S34
11	Glycosidic torsion angles for the modified nucleotides snapshot in Fig. 4 in the main text, and mean / standard deviations over 200.5–1000.5 ns	S34
12	Chemical shift assignment for reduced $^{6NO}\underline{U}$3 duplex, **10**^{NOH}	S40

13	Chemical shift assignment for reduced $^{5NO}\underline{U}3$ duplex, **9^{NOH}**	S41
14	Assignments of exchangeable protons at 10 °C in H_2O for duplexes **10^{NOH}** and **9^{NOH}**	S42
15	Conformation of the $^{NO}\underline{U}3$ residue in the two spin-labelled duplexes	S42
16	Chemical shift assignment for $^{6NO}\underline{U}3$ duplex **10**	S43
17	Chemical shift assignment for $^{5NO}\underline{U}3$ duplex **9**	S44

1. Oligonucleotide synthesis and purification

Standard nucleoside phosphoramidites and additional reagents were purchased from Link Technologies and Applied Biosystems. Oligonucleotides were synthesised with an Applied Biosystems 394 automated DNA/RNA synthesiser, with a standard 1-μmol scale phosphoramidite cycle of acid-catalysed detritylation, coupling, capping and iodine oxidation. All β-cyanoethyl phosphoramidite monomers were dissolved in anhydrous acetonitrile at a concentration of 0.1 M immediately before use. For the 2′-alkynyluridine phosphoramidite, which was synthesised according to the procedure of Haugland *et al.* (see Scheme S1 for a summary of this synthetic route),(1) an extended coupling time (10 min) was used. Cleavage of the oligonucleotides from the solid support and subsequent deprotection was achieved by immersion in concentrated aqueous NH_3 for 1 h at room temperature, followed by heating in a sealed tube for 5 h at 55 °C.

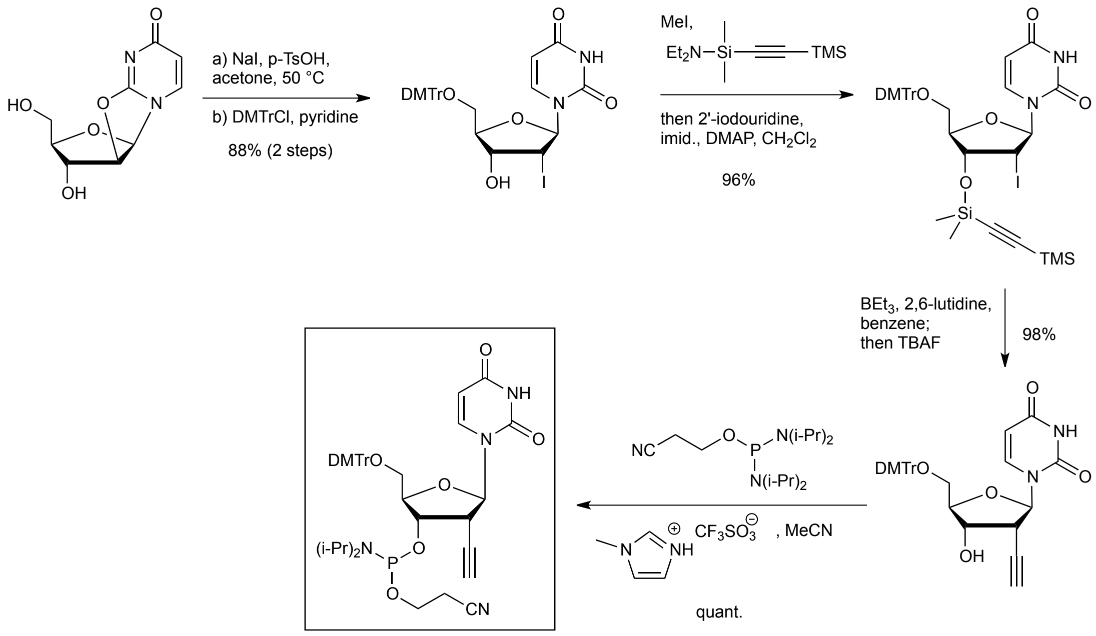

Scheme S1. Synthesis of the 2'-alkynyluridine phosphoramidite used in this work. See Reference 1 for details and procedures.

Spin-labelling of 2′-alkynyl-modified oligonucleotides was performed according to the procedure of Haugland *et al.*(1) Oligonucleotides were purified on an RP-HPLC system (Gilson) with a Luna 10-μm C8 100 Å pore Phenomenex 10 × 250 mm column, with a gradient of acetonitrile in triethylammonium bicarbonate (TEAB) buffer. The buffers were prepared by first diluting a 1 M

stock solution of TEAB to 0.2 M, adjusting its pH to 7-8, then diluting this to 0.1 M with either water (buffer A) or acetonitrile (buffer B).

UPLC-MS characterisation of oligonucleotides was performed on an Acquity UPLC system with a BEH C18 1.7-µm column (Waters) in conjunction with a Waters Xevo G2-QTOF mass spectrometer (ESI^{-} mode). A gradient of methanol in TEA and hexafluoroisopropanol (HFIP) was used (buffer A: 8.6 mM TEA, 200 mM HFIP in 5% methanol/water (vol/vol); buffer B: 20% buffer A in methanol). Buffer B was increased from 0-70% over 8 min, at a flow rate of 0.2 mL min^{-1}. Raw data were processed and deconvoluted with the MassLynx software package. Oligonucleotides were quantified based upon their absorption at 260 nm using the nearest-neighbour molar extinction coefficient.

Table S1. Mass spec and UV melting data for modified oligonucleotides.

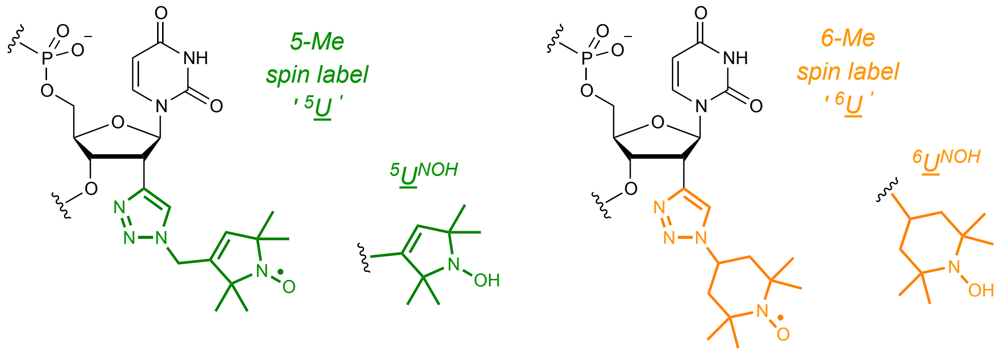

Duplex	Sequence	Mass obs. (calc.)	T_m (°C)[a]
**5**	5′-CGC AAA TT^{5}**U** GCG-3′	3851 (3850)	27.7 ± 1.4[b]
**6**	5′-CGC AAA TT^{6}**U** GCG-3′	3853 (3852)	26.2 ± 1.0[b]
**7**	3′-GCG ^{5}**U**TT TTT CGC-5′	3824 (3824)	39.2 ± 0.3[c]
**9**	5′-CT^{5}**U** ACG CGT CAT TG-3′	4435 (4435)	n/a
**10**	5′-CT^{6}**U** ACG CGT CAT TG-3′	4438 (4437)	n/a
**9^{NOH}**	5′-CT^{5}**U**^{NOH} ACG CGT CAT TG-3′	n/a[d]	n/a[d]
**10^{NOH}**	5′-CT^{6}**U**^{NOH} ACG CGT CAT TG-3′	n/a[d]	n/a[d]

[a] Error = 2 × standard deviation. [ssDNA]$_{total}$ = 6 µM, phosphate buffer (pH 7.0, 10 mM), 200 mM NaCl. [b] T_m of native duplex = 54 °C (2). [c] 1:1 complex with the complementary, non-modified strand. T_m of native duplex = 55 °C (3). [d] Duplex **9^{NOH}** and **10^{NOH}** were prepared in situ for NMR studies.

2. EPR spectroscopy

Sample preparation. EPR samples were diluted from an 800 μM spin-labelled modified-DDD single stranded stock in deuterium oxide containing 150 mM NaCl, 10 mM Na_3PO_4, pH 7.4 and 50% glycerol by volume using the same buffer. Concentrations of 200, 100, 40, 20 and 10 μM ssDNA at 40 or 50 μl was loaded into 3 mm OD suprasil quartz EPR tubes for the 4-pulse DEER experiment.(4,5) EPR tubes were either flash frozen (room temperature, 293 K) in liquid nitrogen, or annealed in the standard freezer (253 K) for several days before removing and immediately freezing in liquid nitrogen, for the results described below as 'pre-cooled'. A Bruker Elexsys E580 high-powered (150 W) Q-band (34 GHz) with an ER 5106QT-2w cylindrical resonator was cooled to 50 K and samples loaded from the liquid nitrogen dewar.

DEER experiments. The 4-pulse DEER experiment(4,5) used $\pi/2$(observer) - $\tau 1$ - π(observer) - t - π(pump) - ($\tau 1$ + $\tau 2$ - t) - π(observer) with the observer sequence (32 ns pulses) at 34 GHz and on the maximum intensity of the nitroxyl echo-detected field sweep and the pump pulse (16 ns) at 80 MHz lower frequency. $\tau 1$ was set at 400 ns and nuclear modulation averaging (5 sets with a difference of 24 ns each time) was used. $\tau 2$ was either 2, 3 or 4 μs and t was stepped in 8 ns increments. DeerAnalysis2016(6) was used to process the data by removing the last 800 ns to ensure no artefacts were present and setting the zero-time to 328 ns before fitting the homogeneous background decay function.(5) Tikhonov Regularisation was used to extract distance distributions using the parameter determined by DeerAnalysis2016 and the L-curve method.

Distance distributions: Throughout the manuscript, distances have been measured from halfway between the N and O atoms of the nitroxide, as is standard in the analysis of DEER data. Modelling was carried out to identify spin density on N and O using Gaussian 09, as follows:

6-Me: UHF/6-31G* // UB3LYP/6-31G*, iop(6/33=2) iop(6/42=6) Pop=MK

Atomic spin densities: largest coefficients found are N (0.440) and O (0.606).

Sum over all atoms: 1.000.

UB3LYP/6-31G*: 0.461 for N and 0.502 for O.

5-Me: UHF/6-31G* // UB3LYP/6-31G*, iop(6/33=2) iop(6/42=6) Pop=MK

Atomic spin densities: largest coefficients found for N (0.489) and for O (0.568).

Sum over all atoms: 1.000. UB3LYP/6-31G*: 0.475 for N and 0.494 for O

Continuous Wave EPR. Samples were prepared as for the DEER experiments, but were instead loaded into capillary tubes. Measurements were taken for the duplex samples prepared from 200 and 10 μM ssDNA at room temperature with a Bruker EMX operating at X-band (9.83 GHz) and a SHQE resonator under non-saturating conditions.

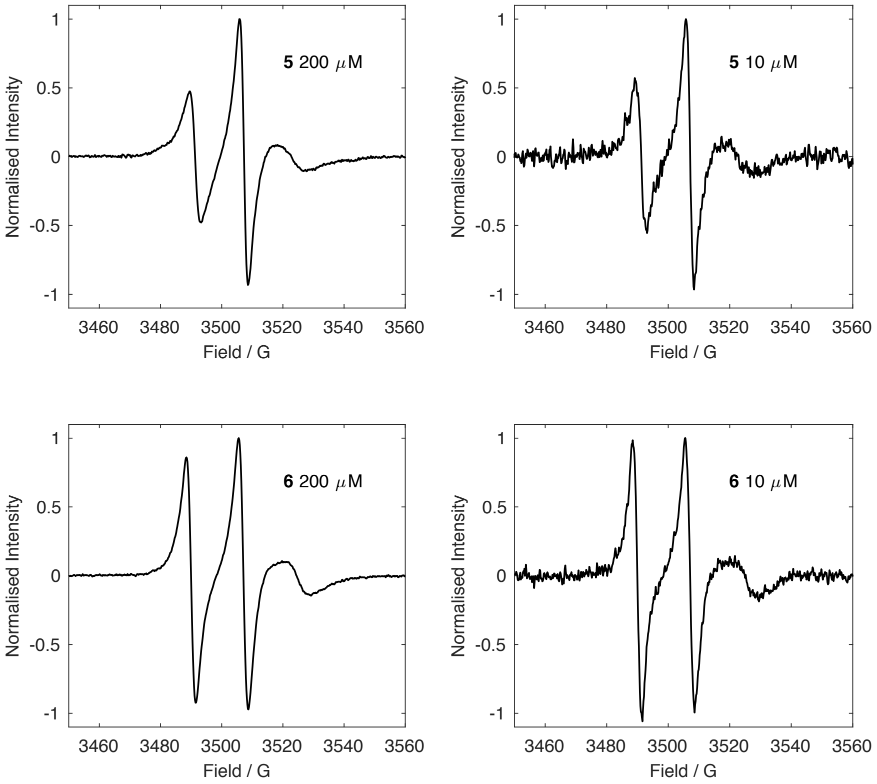

Figure S1: Room temperature CW EPR spectra of the spin-labelled DDD in buffer/glycerol. The concentration given is based on the concentration of (self-complementary) single stranded DNA. There are small differences between duplexes **5** and **6**, but very little change between the concentrations tested.

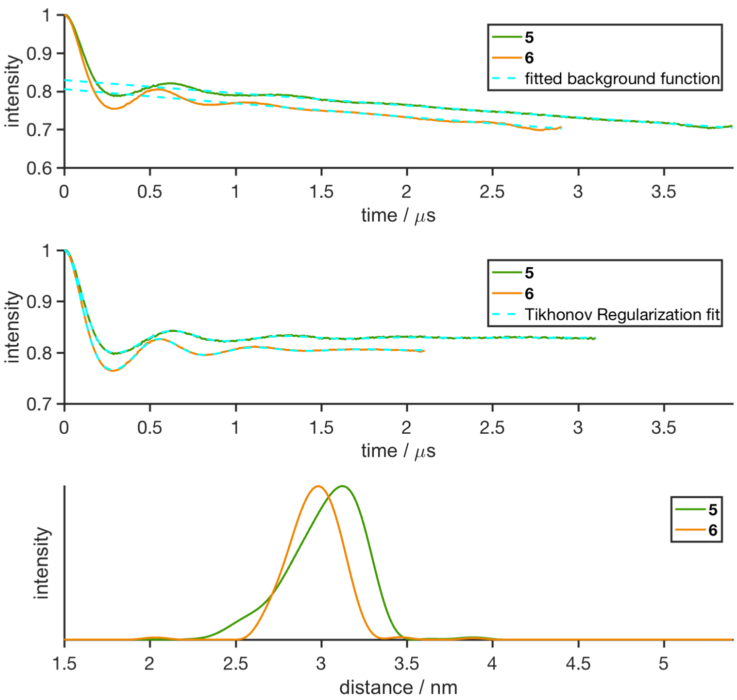

Figure S2: DEER time trace (top, background correction shown in cyan dash), background corrected time trace (middle, distribution fit shown in cyan dash) and distance distributions (bottom) for the spin-labelled duplexes frozen from room temperature with a maximum duplex concentration of 100 μM (200 μM in single stranded DNA). Tikhonov regularisation parameters are 94 and 22 for **5** and **6** respectively. The distance distribution results also shown in main paper.

The modulation depth of the DEER data was found to depend not only on oligonucleotide concentration, but also on the temperature of the sample prior to rapid freezing in liquid nitrogen for loading into the EPR spectrometer (Figure S3); rapid cooling of DNA solutions from 293 K gave smaller modulation depths than when cooled from 253 K where the solution was near freezing, implying less complete duplex formation at the higher temperature (estimated at 54% and 69% for **5** and **6** respectively at 200 μM concentration of ssDNA, cooled from 293 K, compared to 100% when cooled from 253 K, see Figure S4). Importantly, the actual distance distributions remain almost equivalent regardless of the method used for freezing or the concentration of the sample (Figure S5), showing that irrespective of the extent of duplex formation, the nature of the duplex is near identical, and is well-defined.

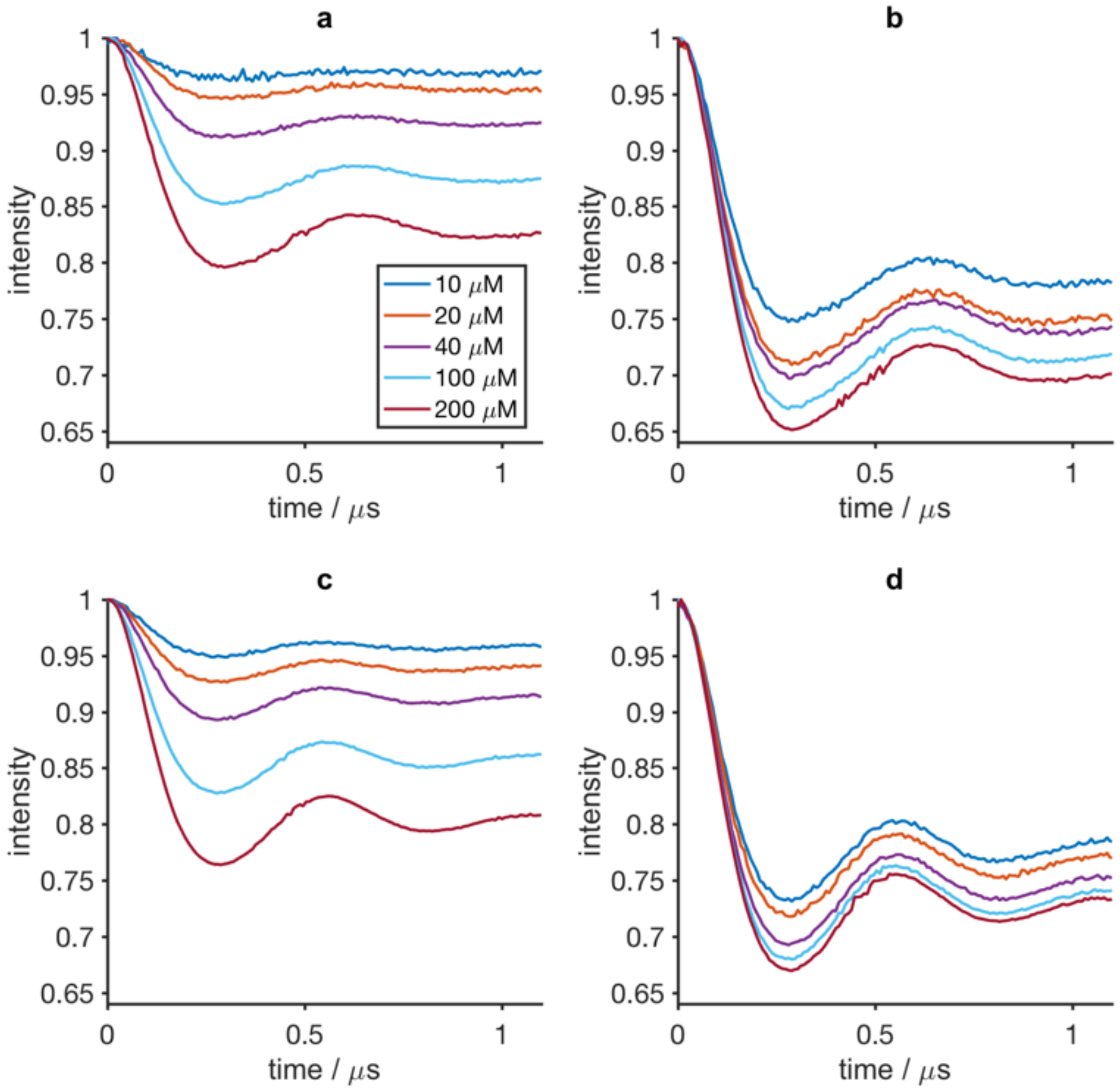

Figure S3: DEER time traces for spin-labelled duplexes **5** (**a** and **b**), **6** (**c** and **d**)) at different concentrations (legend shown in a) and frozen in liquid nitrogen from either 293 K (**a** and **c**) or 253 K (**b** and **d**). All DEER experiments were carried out with a τ_2 of 2 μs and have been background corrected.

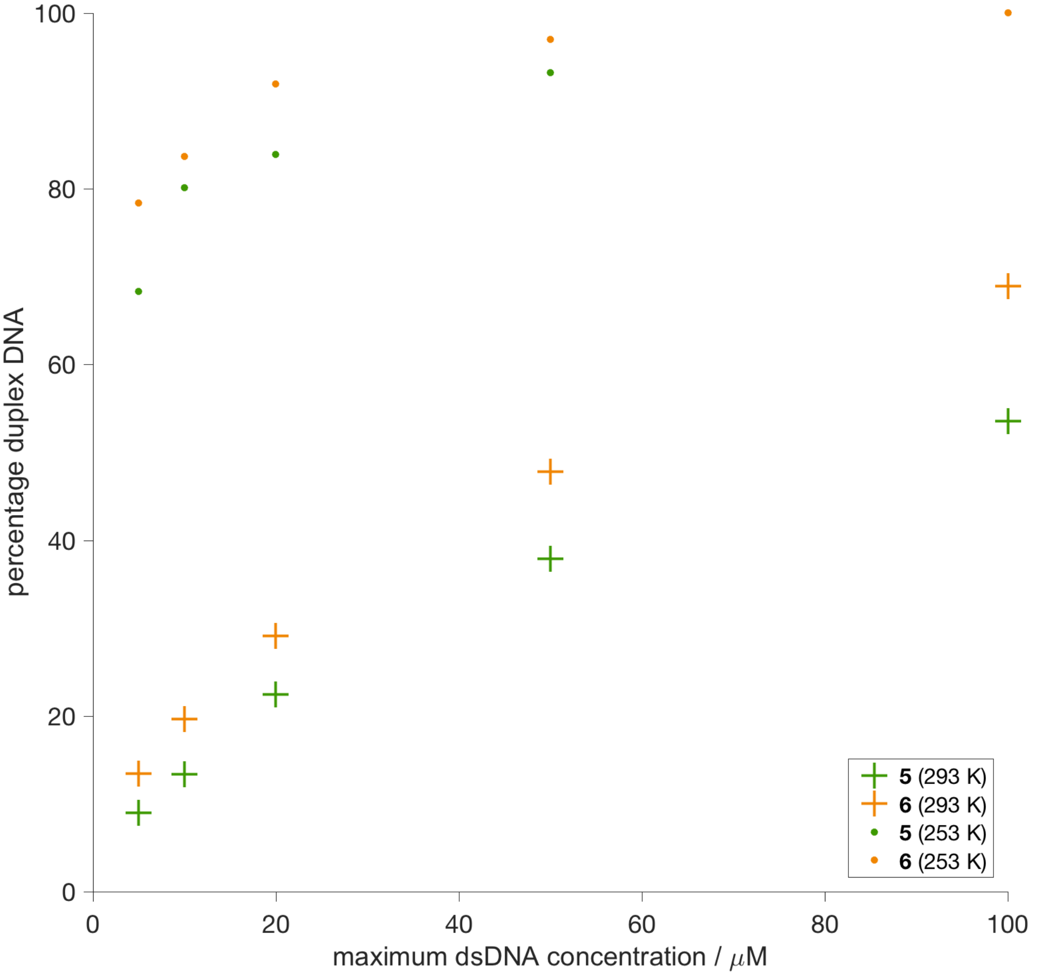

Figure S4: Modulation depths from Figure S3 were used to estimate the percentage duplex formation using the following equation, where the modulation depth Δ for the contribution from spins in the duplex depends on the average number ‹*n*› of spins in the duplex (with *C* as a constant) as:(7)

$$\langle n \rangle = 1 + \frac{\log(1 - \Delta)}{C}$$

To give a value for *C* we use the approximation that the modulation depth for the 200 μM ssDNA samples frozen from 253 K (pre-cooled) gives the maximum dsDNA obtainable under these measurement conditions, for each label (assigned value of 100% dsDNA). We also assume that each strand is fully spin labelled. The percentage of duplex DNA in the mixture, as shown in Figure S4, is given from the above equation as (‹*n*›-1) x 100. The maximum dsDNA concentration at a given value is therefore 0.5 x [ssDNA].

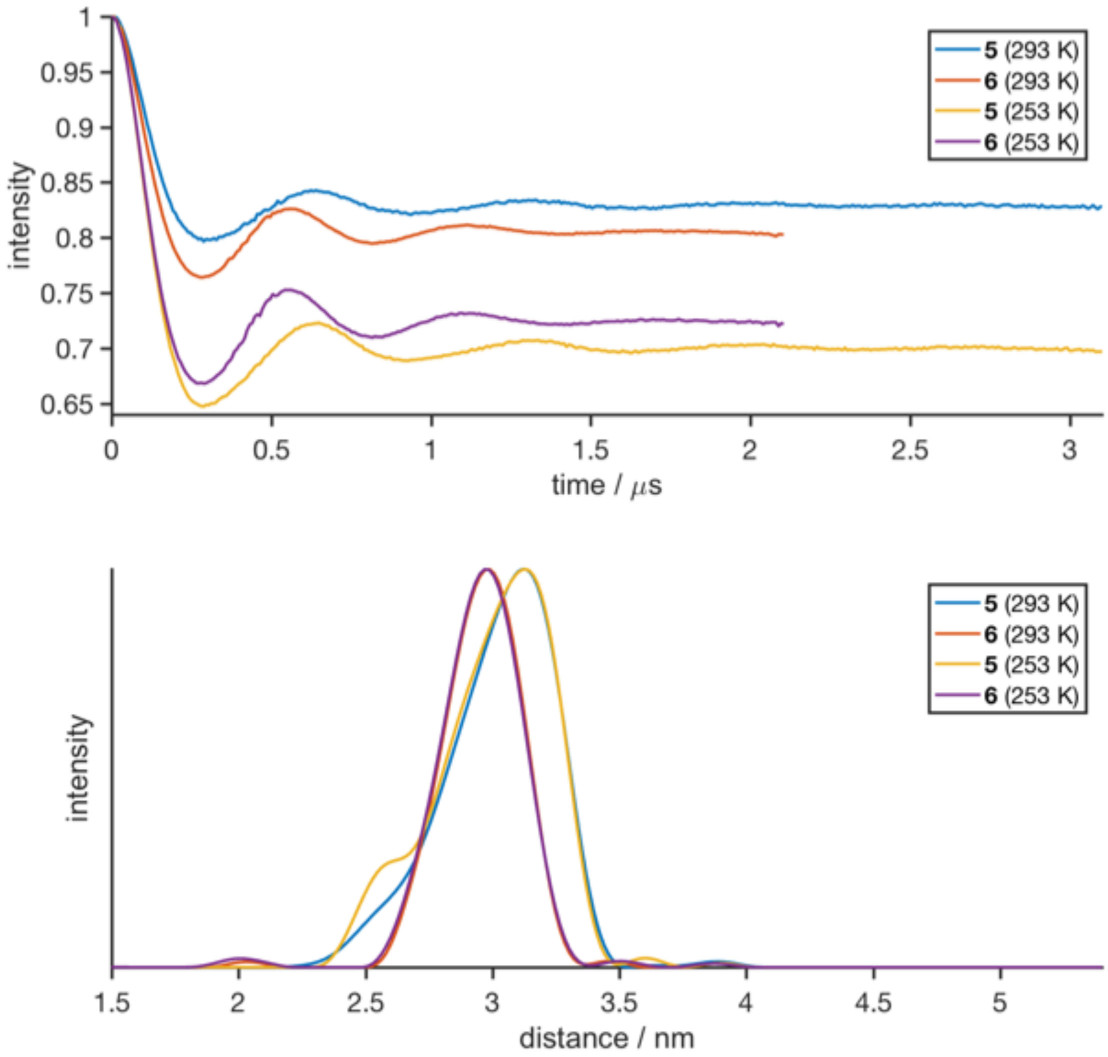

Figure S5: DEER time traces for spin-labelled duplexes with maximum possible duplex concentration of 100 μM (ssDNA 200 μM) for samples frozen from 293 K or 253 K ("pre-cooled"). The DEER time traces (top) have been background corrected. The distance distributions do not differ for **6** regardless of freezing conditions, and only little for **5**. Expansion of the small peak at 3.5 nm for **6** shows a consistent signal irrespective of the mode of cooling. Notably, the certainty of this small peak (See Figure S6) is lower than that of the main peak.

Data Validation:

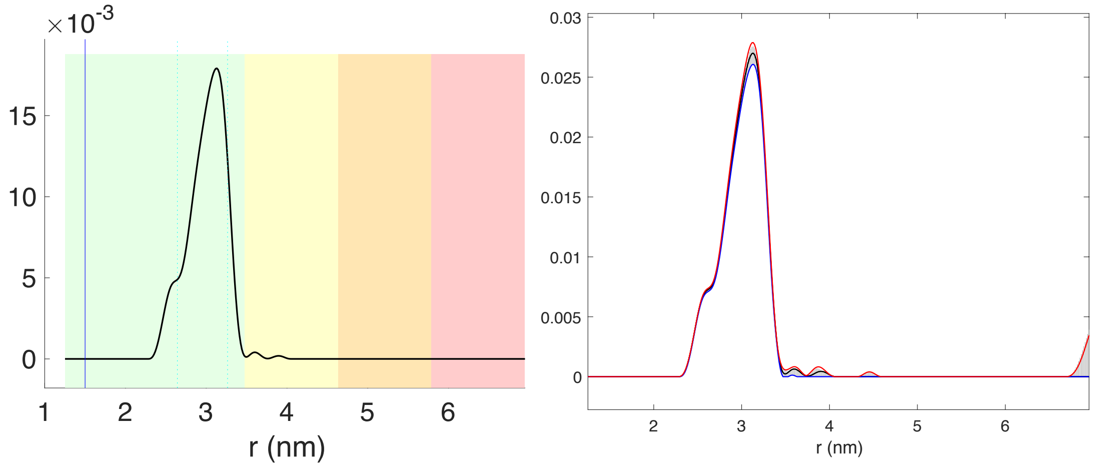

Figure S6. Duplex **5.** Validation of distance certainty with maximum possible duplex concentration of 100 μM (ssDNA 200 μM) frozen from 253 K using DeerAnalysis2016 with Tikhonov Regularisation parameter 55. The left figure shows the certainty range based on the length of the DEER time window and the modulation frequency, green indicates the distribution is well measured. The right figure uses the default values for varying the background-fit start value in a "validation" of the distance distribution. The results show that the main peak and its shoulder are robustly measured.

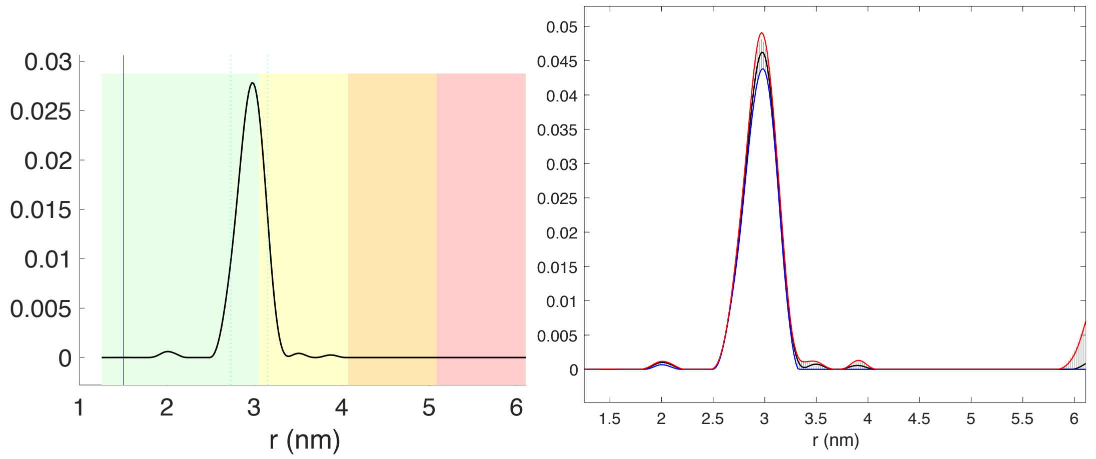

Figure S7. Duplex **6.** Validation of distance certainty with maximum possible duplex concentration of 100 μM (ssDNA 200 μM) frozen from 253 K using DeerAnalysis2016 with Tikhonov Regularisation parameter 22. The left figure shows the certainty range based on the length of the DEER time window and the modulation frequency, green indicates the distribution is well measured. The right figure uses the default values for varying the background-fit start value in a "validation" of the distance distribution. The results show that the main peak is robustly measured**.**

3. X-ray Crystallography

Oligonucleotide crystallization. Duplexes **6** and **7** (**Table S2**) were prepared from the purest HPLC fraction of each oligonucleotide to a concentration of 1 mM duplex in a 10 mM solution of KCl. These stock solutions were used to set up Natrix HT crystallization screens (Hampton Research) in 96-well Greiner plates, using 100 nL DNA solution and 100 nL crystallization reagent solution dispensed by a Microsys instrument (Cartesian Technologies). Plates were sealed and kept at 21 °C in a Formulatrix Storage and Imaging system for 20 days, until suitable crystals had grown. Crystallization conditions are given in **Table S3**.

Diffraction data collection. Crystals were flash-cooled in liquid nitrogen. Diffraction data were collected at 100 K at the Diamond Light Source synchrotron science facility, Harwell, Beamline I04, using Pilatus 6M hybrid pixel array detectors. For the ^{6NO}U and ^{5NO}U substituted duplexes, X-ray wavelengths of 0.979 and 0.916 Å were used, respectively. See **Table S4**.

Diffraction data processing, phase determination, model building, and refinement. Data were indexed and scaled with XDS(8) and AIMLESS.(9) The structures 6QJS (duplex **6**) and 6QJR (duplex **7**) were solved by molecular replacement with Phaser(10) using PDB codes 1S2R and 1D98 as search models. To avoid model bias, the residues corresponding to the modification sites were removed from the search model. Additionally, terminal base pairs from 1S2R had to be removed for molecular replacement of the 6-Me structure to be successful. After an initial stage of refinement with REFMAC5,(11) missing residues or atoms were rebuilt manually with COOT,(12) followed by successive rounds of further refinement.

Analysis of X-ray crystal structures. Inter- and intra-base-pair parameters, puckering and torsion angles were determined using 3DNA.(13) Groove dimensions were determined using Curves+.(14) RMSDs were determined using PyMOL. Ideal DNA duplexes were generated using w3DNA.(15) Interspin distances were determined using UCSF Chimera.(16)

Table S2. Sequences of oligonucleotides used for crystallization.

PDB code	DNA duplex sequence
6QJS	**6** d(CGCAAATT^{6Me}UGCG)$_2$
6QJR	**7** d(CGCTTTTT^{5Me}UGCG)/ d(CGCAAAAAAGCG)

Table S3. Conditions for oligonucleotide crystallization.

**Duplex**	**6 d(CGCAAATT^{6Me}UGCG)$_2$**	**7 d(CGCTTTTT^{5Me}UGCG)/ d(CGCAAAAAAGCG)**
**DNA duplex (mM)**	1	1
**Potassium chloride (mM)**	10	90
**Sodium chloride (mM)**	80	0
**Magnesium chloride hexahydrate (mM)**	20	20
**Sodium cacodylate trihydrate (mM)**	40 (pH 5.5)	40 (pH 6.0)
**(±)-2-Methyl-2,4-pentanediol (% v/v)**	35	45
**Hexammine cobalt(III) chloride (mM)**	2	0
**Spermine tetrahydrochloride (mM)**	0	12

Table S4. X-ray data collection statistics.

**Data set**	**Resolution (Å)**[a]	**X-ray wave-length (Å)**	**Space group and unit cell dimensions (Å)**	**Reflections measured / unique**	**R_{meas} (%)**[b]	**I/σI**	**Complete-ness (%)**	**Half-set correlation $CC_{1/2}$**
**6** (6QJS)	37.2-1.80 (1.84-1.80)	0.979	$P3_221$<br>a = b= 43.0, c = 97.5	37913/ 9926	9.2 (73.9)	9.3 (2.6)	97.6 (95.1)	0.994 (0.684)
**7** (6QJR)	35.1-2.90 (3.10-2.90)	0.916	$P2_12_12_1$<br>a = 25.3, b= 41.2, c = 67.0	5768/ 1731	5.7 (18.4)	10.3 (2.7)	99.0 (99.6)	0.999 (0.998)

[a] Values in parentheses are for the highest-resolution shell.

[b] Rmeas is a redundancy independent R-factor as defined in Diedrichs & Karplus, 1997.(17)

Table S5. Twinning statistics for duplex **6** (6QJS).

Twin domain	Twin operator	Twin fraction
1	h,k,l	0.481
2	-h,-k,l	0.519

Table S6. Refinement statistics for duplexes **6** and **7** (6QJS and 6QJR).

Data set	Resolution (Å)	Total no. of non-hydrogen atoms	No. of water molecules	R-factor (%) / N reflections	R_{free} (%) / N reflections	*R.m.s. deviations*	
						Bond lengths (Å)	Bond angles (degrees)
**6**	37.2-1.8	519	7	21.3 / 9365	25.1 / 555	0.014	1.67
**7**	35.1-2.9	490	0	18.8 / 1621	24.4 / 96	0.008	1.16

Table S7. Phase angles and glycosyl torsion angles for duplexes **6** and **7**.

Duplex / nt	X-ray phase angle	Sugar conformation	X-ray glycosyl torsion angle
**6** / $9^{6}\underline{\text{U}}$	49°	C4'-*exo*	-141°
**6** / $21^{6}\underline{\text{U}}$	36°	C3'-*endo*	-146°
**7** / $9^{5}\underline{\text{U}}$	45°	C4'-*exo*	-146°

Table S8. Sugar puckers and pseudorotation phase angles of crystal structures PDB 6QJS (containing 6-Me-labelled uridine), PDB 6QJR (containing 5-Me-labelled uridine), and those of their unmodified counterparts PDBs 1S2R and 1D98, respectively. Pseudorotation phase angles are shown in parentheses.

	**6QJS (X = 6-Me)**		**1S2R**	
Base pair	Strand A	Strand B	Strand I	Strand II
2: G·C	C3'-*exo* (187.6°)	C2'-*endo* (160.4°)	C2'-*endo* (150.9°)	C2'-*endo* (166.6°)
3: C·G	O4'-*endo* (83.1°)	C2'-*endo* (159.2°)	C4'-*exo* (60.3°)	C1'-*exo* (139.9°)
4: A·**X**	C2'-*endo* (163.6°)	**C4'-*exo* (48.8°)**	C2'-*endo* (170.8°)	C2'-*endo* (145.7°)
5: A·T	C3'-*exo* (183.3°)	C2'-*endo* (147.7°)	C1'-*exo* (128.5°)	C1'-*exo* (138.4°)
6: A·T	C2'-*endo* (146.1°)	C1'-*exo* (108.7°)	C2'-*endo* (147.6°)	C1'-*exo* (121.3°)
7: T·A	C1'-*exo* (110.4°)	C2'-*endo* (144.7°)	O4'-*endo* (95.8°)	C1'-*exo* (139.4°)
8: T·A	C2'-*endo* (150.1°)	C2'-*endo* (156.1°)	C1'-*exo* (139.6°)	C2'-*endo* (161.9°)
9: **X**·A	**C3'-*endo* (35.5°)**	C2'-*endo* (159.5°)	C1'-*exo* (141.0°)	C2'-*endo* (168.7°)
10: G·C	C2'-*endo* (152.3°)	O4'-*endo* (83.5°)	C2'-*endo* (144.0°)	O4'-*endo* (77.0°)
11: C·G	C2'-*endo* (156.3°)	C3'-*exo* (193.9°)	C2'-*endo* (166.3°)	C2'-*endo* (155.4°)

	**6QJR (X = 5-Me)**		**1D98**	
Base pair	Strand I	Strand II	Strand I	Strand II
1: C·G	C2'-*endo* (161.9°)	C4'-*exo* (53.9°)	C2'-*endo* (176.8°)	C3'-*endo* (4.3°)
2: G·C	C3'-*endo* (15.6°)	C4'-*exo* (43.4°)	C4'-*exo* (68.8°)	C4'-*endo* (236.2°)
3: C·G	O4'-*endo* (77.9°)	C2'-*endo* (144.9°)	O4'-*endo* (102.6°)	C2'-*endo* (161.9°)
4: A·**X**	C2'-*endo* (165.6°)	**C4'-*exo* (45.3°)**	C3'-*exo* (206.2°)	C1'-*exo* (109.8°)
5: A·T	C2'-*endo* (173.9°)	C4'-*exo* (65.8°)	C2'-*endo* (158.8°)	C2'-*endo* (158.3°)
6: A·T	C2'-*endo* (167.3°)	C1'-*exo* (141.4°)	C2'-*endo* (149.7°)	C1'-*exo* (131.5°)
7: A·T	C2'-*endo* (172.6°)	C2'-*endo* (156.5°)	C1'-*exo* (127.4°)	C3'-*exo* (205.1°)
8: A·T	C2'-*endo* (161.8°)	C2'-*endo* (152.6°)	C2'-*endo* (158.5°)	C2'-*endo* (146.6°)
9: A·T	C2'-*endo* (171.9°)	C2'-*endo* (154.5°)	C3'-*exo* (183.7°)	C3'-*exo* (194.4°)
10: G·C	C2'-*endo* (153.7°)	C4'-*exo* (56.9°)	C3'-*exo* (195.9°)	C3'-*endo* (17.7°)
11: C·G	C2'-*endo* (179.7°)	C2'-*endo* (161.8°)	C2'-*endo* (173.5°)	C2'-*endo* (148.7°)
12: G·C	C3'-*endo* (28.9°)	C2'-*endo* (166.3°)	C1'-*exo* (119.7°)	C3'-*exo* (186.8°)

Table S9. χ torsion angles of crystal structures PDB 6QJS (containing 6-Me-labelled uridine), PDB 6QJR (containing 5-Me-labelled uridine), and those of their unmodified counterparts PDBs 1S2R and 1D98, respectively.

	**6QJS**		**1S2R**	
Base pair	Strand A	Strand B	Strand A	Strand B
2: G·C	-93.8	-104.5	-102.1	-115.9
3: C·G	-136.1	-95.5	-131.8	-82.5
4: A·**X**	-76.2	**-145.6**	-97.7	-112.8
5: A·T	-95.4	-109.7	-117.8	-119.1
6: A·T	-101.7	-129.3	-111.4	-124.5
7: T·A	-122.3	-104.2	-128.7	-113.0
8: T·A	-105.9	-105.1	-117.6	-114.3
9: **X**·A	**-140.9**	-82.2	-114.5	-101.9
10: G·C	-98.3	-123.5	-86.2	-129.5
11: C·G	-108.8	-97.1	-102.5	-120.1

	**6QJR**		**1D98**	
Base pair	Strand A	Strand B	Strand A	Strand B
C·G	-122.5	-147.6	-65.1	-136.9
G·C	-158.3	-144.2	-144.7	-117.2
C·G	-133.1	-112.4	-144.3	-69.3
A·**X**	-100.7	**-144.2**	-87.6	-113.7
A·T	-94.3	-103.3	-107.9	-117.5
A·T	-101.2	-145.5	-104.6	-125.5
A·T	-91.3	-102.0	-111.1	-67.3
A·T	-111.3	-127.2	-99.5	-114.5
A·T	-107.5	-119.2	-96.2	-91.2
G·C	-77.6	-165.0	-69.8	-127.8
C·G	-86.6	-77.1	-103.3	-122.0
G·C	-88.9	-117.1	-113.6	-71.5

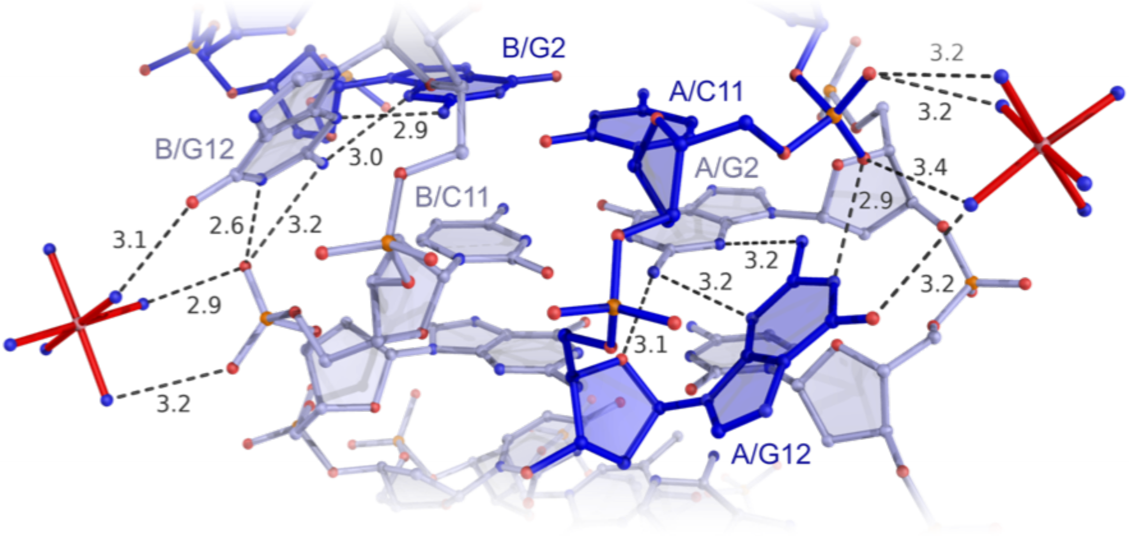

Figure S8. The terminal base pairs of Duplex **6** (dark blue) are disrupted by crystal-packing interactions with cobalt(III) hexammine ions and symmetry mates (grey).

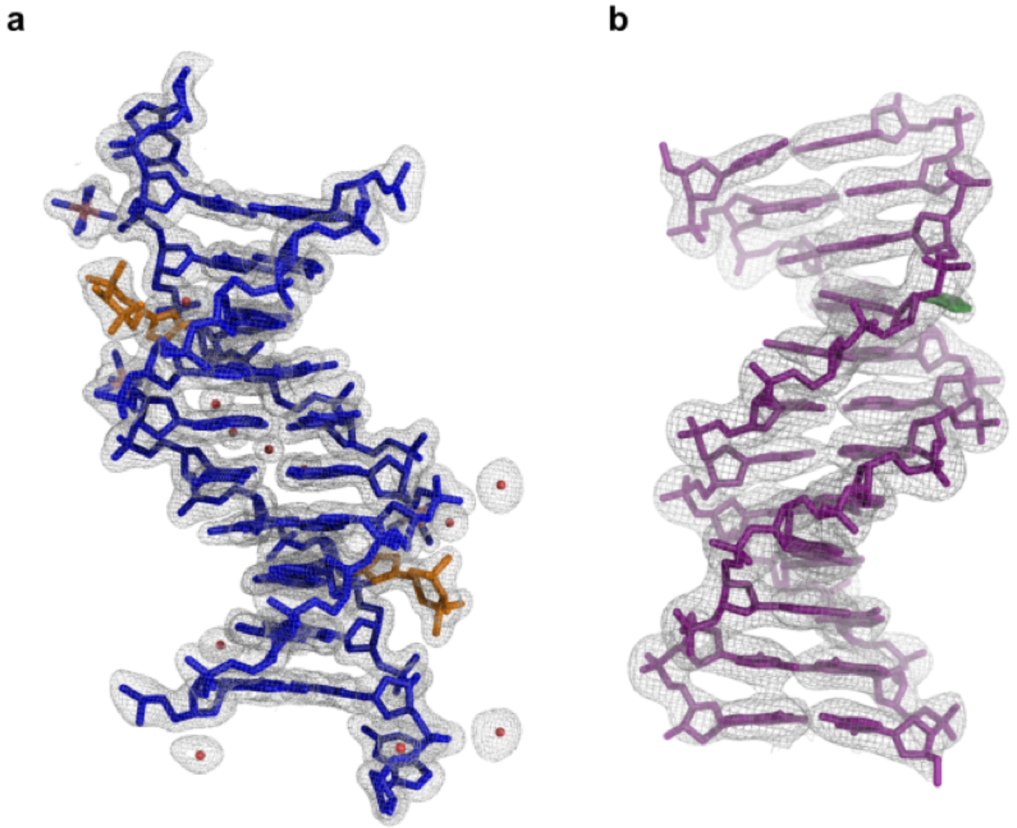

Figure S9. $2F_o$-F_c electron density maps of spin-labelled DNA duplexes, contoured at 1.0 σ. **a** 6QJS (blue, containing 6-Me). Electron density is observed for both spin labels (orange) and all residues except dC1. **b** 6QJR (purple, containing 5-Me). Electron density is observed for all residues except the spin label, for which only density corresponding to the triazole group (green) can be seen.

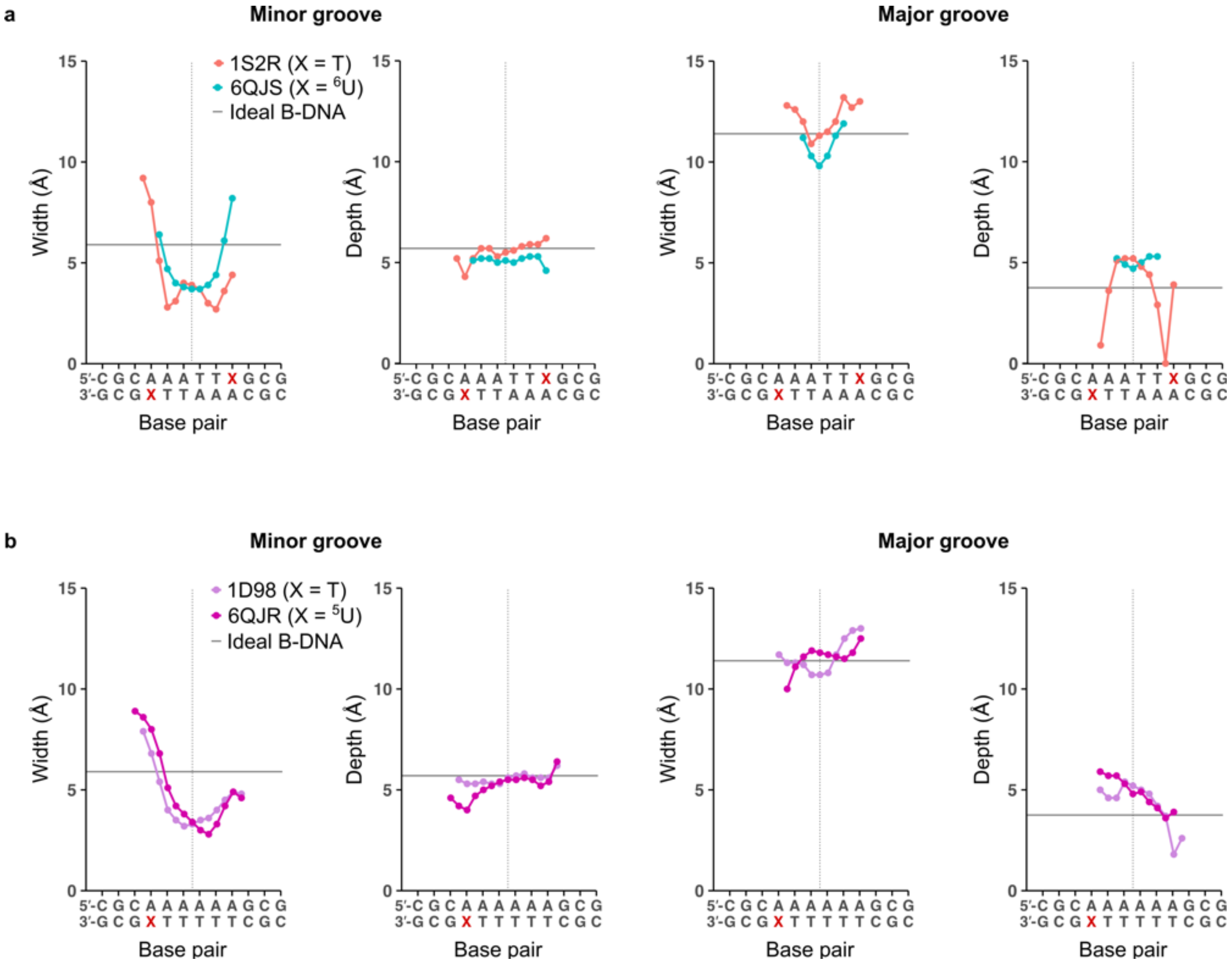

Figure S10. Groove dimensions of spin-labelled DNA duplexes and their unmodified counterparts. Since both spin-labelled duplexes crystallized in different space groups to their unmodified analogues, differences in groove dimensions may be attributed in part to differences in crystal packing. **a** Groove dimensions of the 6-Me-labelled duplex 6QJS, its unmodified counterpart 1S2R, and ideal B-DNA.(15) **b** Groove dimensions of the 5-Me-labelled duplex 6QJR, its unmodified counterpart 1D98, and ideal B-DNA.

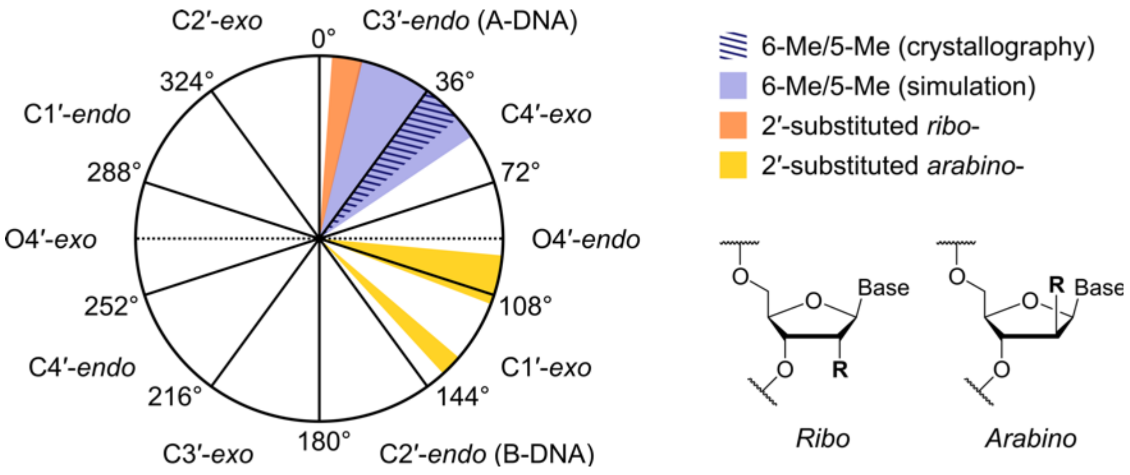

Figure S11. Pseudorotation wheel, showing the ranges of phase angles of the 2′-substituted nucleotides from all eleven B-DNA crystal structures containing such modifications deposited in the PDB at the time of writing. Also shown are the ^{6}U and ^{5}U phase angles determined in this study by X-ray crystallography and MD simulations. Excluding the spin-labelled structures, there are currently only two examples of B-DNA structures containing 2′-substituted *ribo*nucleotides (PDB codes 3EY3 and 2DPC). In total, these two structures contain 10 symmetrically inequivalent modified nucleotides, which all adopt A-DNA-like phase angles of 4-14° (C3′-*endo*). By contrast, the phase angles of the 2′-substituted arabinonucleotides from the other 9 structures (20 unique nucleotides, PDB codes 4KW0, 2FIH, 461D, 436D, 1DPN, 478D, 460D, 389D, 388D) are all more B-like, clustering into two groups whose ranges are 95-111° and 131-138° (O4′-*endo* and C1′-*exo*). The phase angles of the 6-Me and 5-Me-labelled sugars were found to be somewhat intermediate by both crystallography (36-49°) and simulations (13-56°)—though they were significantly closer to those of the other ribonucleotides, as would be expected.

4. Molecular dynamics simulations

Computational details. All-atom, explicit solvent MD simulations of duplexes **6** (DDD with 6-Me covalently attached to U9 and U21 (^{6}**U**)), **5** (DDD with 5-Me in positions 9 and 21 (^{5}**U**)) and the unlabeled DDD sequence **1** $(CGCAAATTTGCG)_2$ (identical to PDB 1S2R) were performed with Amber 16(18) and Amber 18,(19) using A- and B-DNA conformations of each duplex as starting structures (with exception of the unmodified DDD sequence **1**, where only the B-form was used). Parameter derivation, system set-up and the simulations were performed following a protocol very similar to the one established previously,(20,21) therefore only a brief summary is given here.

We started from two different DNA conformations (A- and B-DNA, both for the duplexes **5** and **6**) because we did not wish to bias the modelling in terms of adopting an A- or B-conformation. Attaching the spin labels at the 2' position of A- and B-DNA has the practical consequence that the labels are located on different sides of the backbone in the two cases, as strain-free attachment at the 2’ position of the sugar is only possible on the outside of the duplex, with the spin label pointing into solution. After MD equilibration, these two different spin label positions end up in the “minor groove conformation” (starting from A-form) and in the “antiparallel conformation” (starting from B-form) respectively, which cannot interconvert in the simulations (see below).

In the simulation where the label was attached to A-DNA as a starting structure, the DNA conformation immediately changed to the B form, as expected and previously reported (for a different system),(22) with the spin label positioned in the minor groove. In the simulation starting from B-DNA, the conformation of the DNA itself remained stable, and the spin label remained in the "antiparallel" conformation, which was more or less identical to the starting conformation. In summary, the same DNA conformation is consistently observed, but with two possible conformations for the spin labels (in the minor groove and antiparallel). These two conformations cannot interconvert in the MD simulations, as the spin labels are on different sides of the backbones in the two cases. Interconversion would require pulling the spin labels through the interior of the duplex, or duplex dissociation, both processes that cannot be achieved in conventional MD simulations.

The OL15 force field (ff99(23,24) + bsc0(25) + ε/ζOL1(26)+χOL4(27)+βOL1)(28) was used for the DNA part, RNA.OL3 (ff99(23,24) + bsc0(25) + χOL3)(29) for the modified uridines, and for the spin labels we used GAFF(30,31) (version 2.1) with RESP charges,(32,33) (http://ambermd.org/tutorials/advanced/tutorial1/section1.htm) based on calculations (UHF/6-31G*//UB3LYP/6-31G*)(34-44) with Gaussian 09,(45) in agreement with the Amber force fields.(23,32) (http://ambermd.org/tutorials/advanced/tutorial1/section1.htm) Parameters describing interactions at the border between the part described by the nucleic acid force field and the GAFF part were taken from GAFF.

The DNA-triazole conjugates were constructed from the A-DNA and B-DNA oligonucleotides (5'-CGCAAATTTGCG-3')$_2$ obtained with NAB (fd_helix) from the AmberTools suite(46) and from the preoptimized (Gaussian 09,(45) UHF/6-31G*//UB3LYP/6-31G*(34-44)), capped (–O-PO_2-O-Me) spin labels, and were initially minimized with implicit solvent (GB/SA) for 100 steepest descent and 400 conjugate gradient steps. The systems were then solvated in truncated octahedra of SPC/E water,(47) exceeding the solutes by 15 Å (simulations starting from B-DNA) and 18 Å, respectively (simulations starting from A-DNA). Sodium counterions and additional NaCl were added (total Na^+ concentration: 150 mM, Joung-Cheatham ion parameters).(48) Visualizations of structures very close to the initial structures (after geometry optimization and heat-up with restraints) and of the cluster representatives with a NO• - NO• distance closest to the experimental maximum (or, to the shoulder that corresponds to the experimental maximum in the case of **5**) are given in Supporting Information Figures S12 – S15. After initial geometry optimization with weak restraints (50 kcal mol^{-1} $Å^{-2}$) on the spin labels and DNA, (5,000 steps; switch from steepest descent to conjugate gradient minimization after 500 steps), 5,000 optimization steps without restraints followed.

The optimized structures were used as starting geometries for Langevin dynamics simulations using pmemd.cuda on Nvidia Tesla K40m graphics cards, using a 2 fs timestep, a collision frequency of 2 ps^{-1}, and SHAKE constraints on bonds involving hydrogen.(49) Periodic boundary conditions were used throughout and the distance cutoff for all nonbonding interactions was set to 10 Å. Long-range electrostatics were described by the particle-mesh Ewald method.(50,51) For van der Waals interactions beyond those included in the direct sum, a continuum model correction

for energy and pressure was used, as implemented in Amber. Simulation snapshots were saved every 100 ps.

System heat-up to 298 K was performed during a 500 ps simulation with weak restraints (10 kcal mol^{-1} $Å^{-2}$) on the spin labels/DNA in the NVT ensemble. After that, 1 μs NPT simulation was performed for each simulation system at 298 K and 1 bar (weak pressure coupling, isotropic position scaling, pressure relaxation time 2 ps) without any restraints. Analyses of the MD data were performed using cpptraj(52) from the Amber(18,19) suite, and vmd 1.9.3(53) and pymol(54) were used for visualization. Standard MD analyses (RMSD, RMSF, radius of gyration, Watson-Crick base pairing/fraying of termini) were performed using cpptraj from the AmberTools suite(52) and the simulations were found to be well equilibrated after 200.5 ns. This initial phase was considered as equilibration phase. Fraying of the terminal base pairs was practically not observed. NO• - NO• distances (calculated from the centers of the NO-bonds, as the spin densities obtained from the Gaussian calculations were almost evenly distributed over N and O, see EPR section for details), the phase angles describing sugar puckering(55) and glycosidic torsion angles χ (O4'-C1'-N1-C2) were analyzed more deeply in the production interval of 200.5-1000.5 ns. The representative structures shown in Figure 4 in the main text were selected using a cluster analysis (hierarchical agglomerative, average-linkage algorithm) of the NO• - NO• distances with cpptraj.(52) Here, the simulation snapshot representing the centroid of the cluster with an NO• - NO• distance closest to the maximum of the calculated distance distribution was selected as representative structure and used for visualization in Figure 4 in the main text.

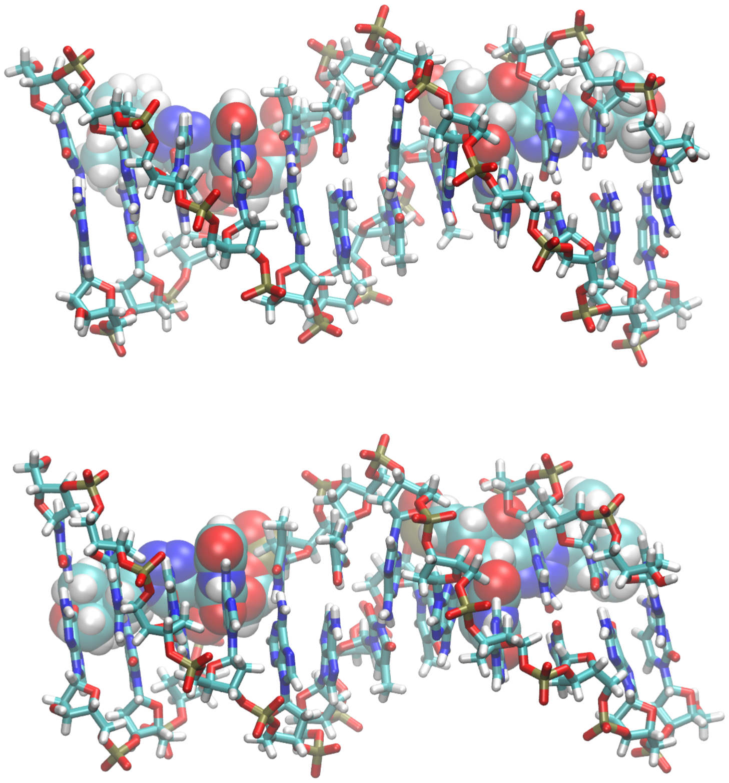

Figure S12. Simulation of system **6**, starting from B-DNA (first of three independent runs): Above: structure at 0.6 ns (very close to starting structure), below: cluster representative of the cluster closest to the maximum of the <u>NO</u>• - <u>NO</u>• distance distribution (distance in this snapshot: 3.34 nm). This figure was created using VMD.(53) (http://www.ks.uiuc.edu/Research/vmd/)

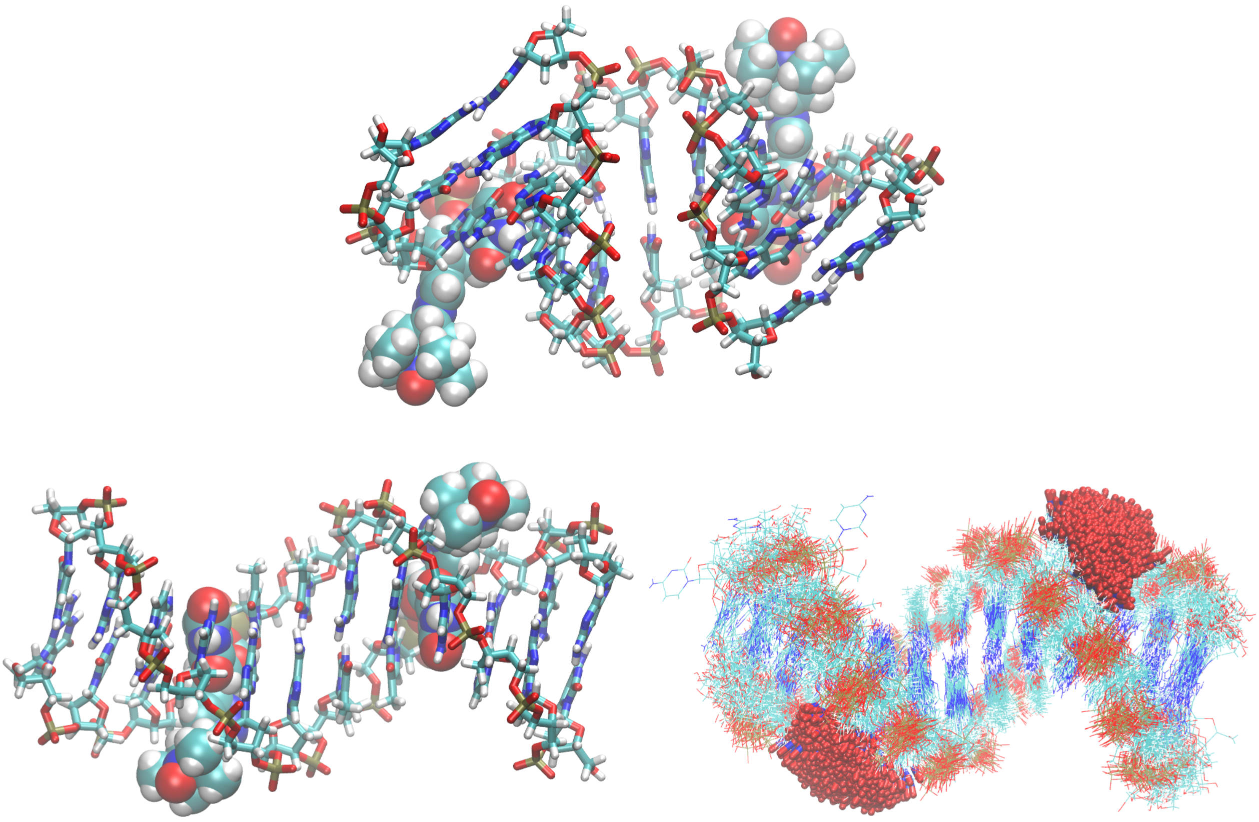

Figure S13. Simulation of system **6**, starting from A-DNA (first of three independent runs): Above: structure at 0.6 ns (very close to starting structure), below, left: cluster representative of the cluster closest to the maximum of the NO• - NO• distance distribution (distance in this snapshot: 2.89 nm), below, right: ensemble of structures over 200.5-1000.5 ns (sticks: NO conformations, each frame shown, lines: whole system, every 100th frame shown). This figure was created using VMD.(53) (http://www.ks.uiuc.edu/Research/vmd/) Note that both the DNA and nitroxide show significant conformational mobility across the simulation.

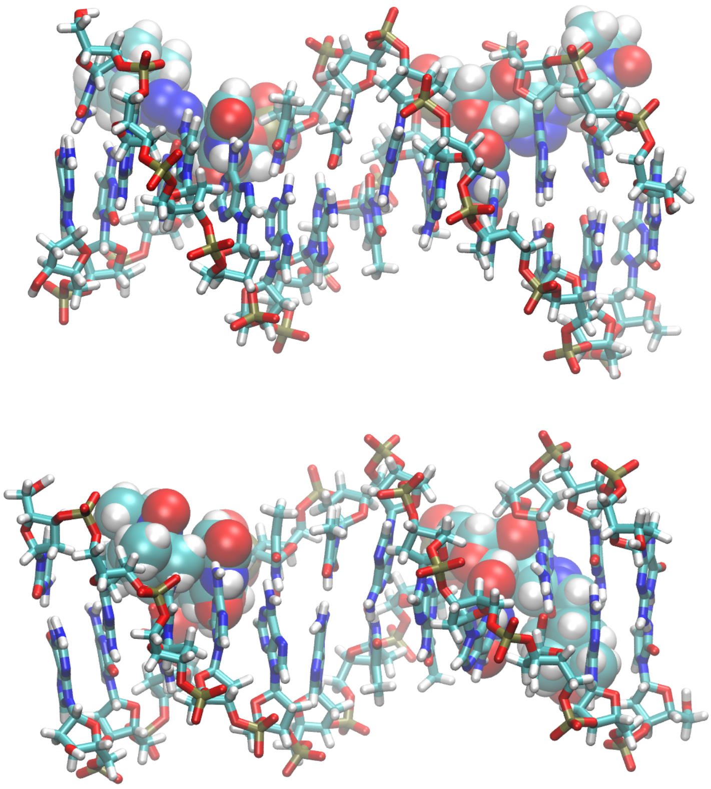

Figure S14. Simulation of system **5**, starting from B-DNA (first of three independent runs): Above: structure at 0.6 ns (very close to starting structure), below: cluster representative of the cluster closest to the maximum of the NO• - NO• distance distribution (distance in this snapshot: 2.76 nm). This figure was created using VMD.(53) (http://www.ks.uiuc.edu/Research/vmd/)

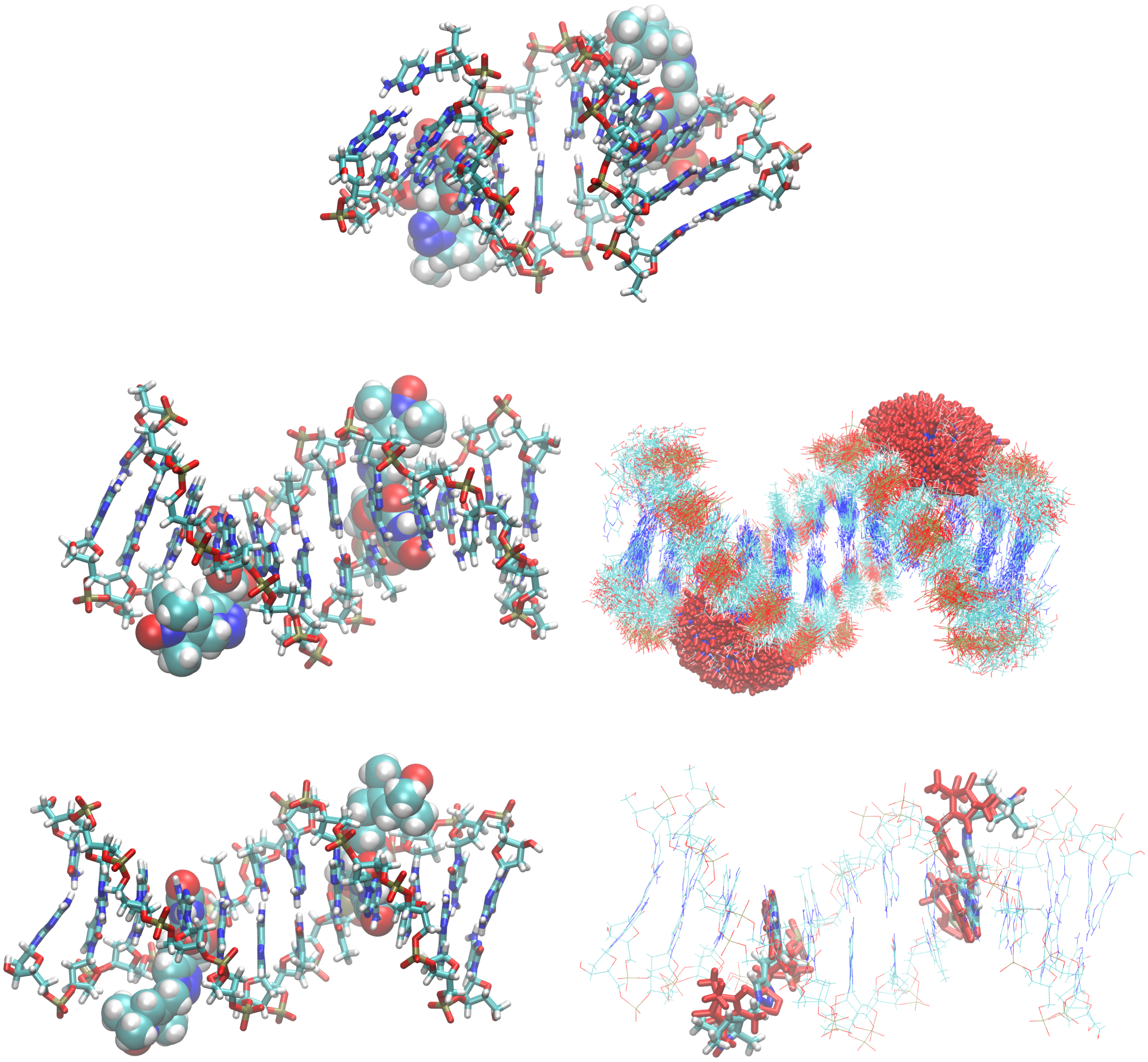

Figure S15. Simulation of system **5**, starting from A-DNA (first of three independent runs): Above: structure at 0.6 ns (very close to starting structure), middle, left: cluster representative of the cluster closest to the maximum of the simulated NO• - NO• distance distribution (distance in this snapshot: 2.99 nm), middle, right: ensemble of structures over 200.5-1000.5 ns (sticks: NO conformations, each frame shown, lines: whole system, ever 100th frame shown), below, left: cluster representative of the cluster closest to the experimental maximum of the NO• - NO• distance distribution (distance in this snapshot: 3.21 nm), below, right: superimposition of cluster representative of the cluster closest to the maximum of the simulated NO• - NO• distance distribution (red) and cluster representative of the cluster closest to the experimental maximum of the NO• - NO• distance distribution (CPK colors). This figure was created using VMD.(53) (http://www.ks.uiuc.edu/Research/vmd/)

In addition to the data shown in Figure 4 in the main text, we have performed two independent repeats of the minor groove simulations of **6** and **5**, with different initial velocities for each of the three runs. The results are shown in Supporting Information Figures S16 and S17. The three simulated spin label distance distributions are near identical for the (more rigid) spin label **6-Me**, and exhibit a larger variation for spin label **5-Me**, which is more flexible due to the additional CH_2 group. This underlines that the more rigid spin label **6-Me** is better suited for comparisons of simulations and DEER experiments than the flexible spin label **5-Me**.

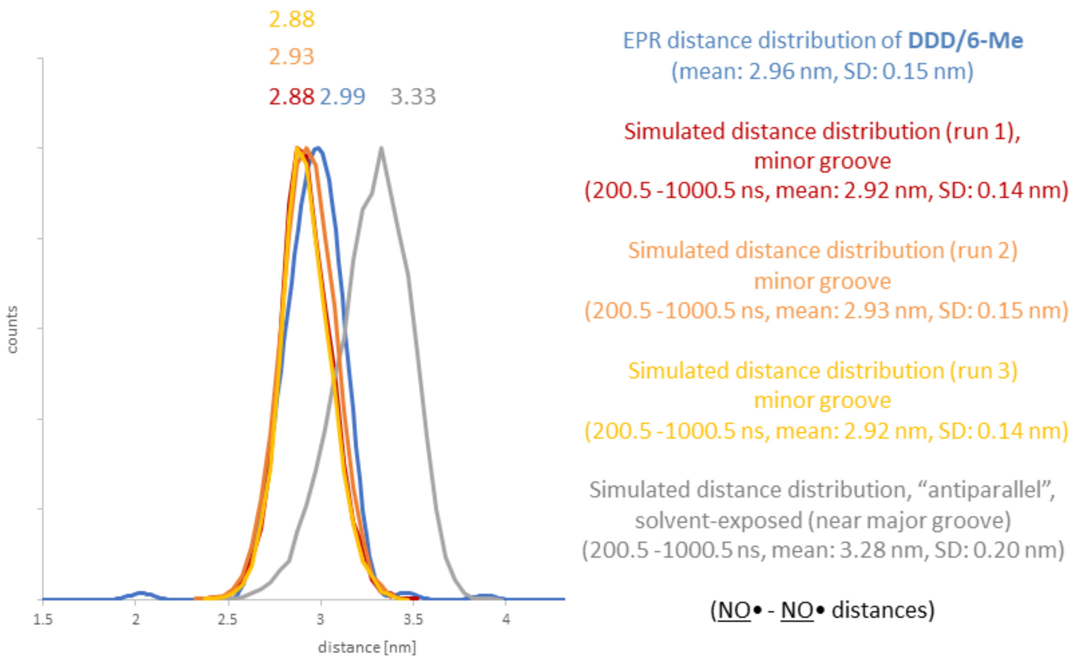

Figure S16. Three independent runs of system **6**, minor groove conformation (simulations starting from A-DNA). Distributions of simulated NO• - NO• distances vs. experiment.

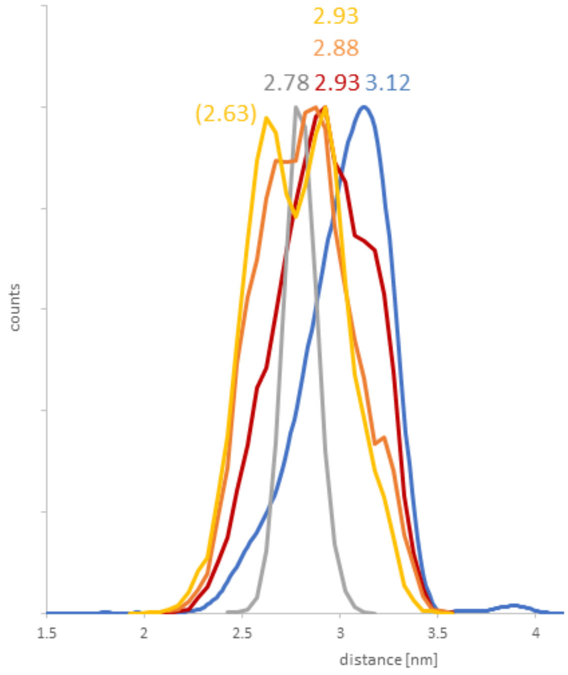

Figure S17. Three independent runs of system **5**, minor groove conformation (simulations starting from A-DNA). Distributions of simulated NO• - NO• distances vs. experiment.

In addition to measuring the NO• - NO• distances, we have performed distance measurements between the spin label atoms (C11) directly attached to the sugar in the position corresponding to O2' in ribose to investigate whether the structural variation of the NO• - NO• distances mainly results from the movements of the spin labels themselves, or from DNA flexibility. In the simulation of system **6** in the minor groove conformation (first run of three) we find a mean NO• - NO• distance of 2.92 ± 0.14 nm and a mean distance of the C11 atoms in the two spin labels of 2.01 ± 0.07 nm. These values indicate that the distance distribution arises not only from mobility of the spin label, but also (to a lesser extent) the duplex itself. The situation is similar for the more flexible spin label in system **5**, where we find a mean NO• - NO• distance of 2.91 ± 0.24 nm and a mean distance of the C11 atoms in the two spin labels of 1.99 ± 0.10 nm.

Analysis of sugar puckers and glycosidic torsion angles. Plots of the mean value of the phase angles describing the sugar pucker(55) of all residues vs. time are shown in Supporting Information Figures S18 and S20 for the first of three independent runs of the simulations of systems **6** and **5**, respectively, each with the spin labels in the minor groove. Phase angles of each DNA residue (mean values over 200.5 - 1000.5 ns) versus sequence are shown in Figures S19 and S21 for duplexes **6** and **5**, also in their minor groove conformations. Tables S10 and S11 give an overview of the sugar pucker phase angles and the glycosidic torsion angles (O4'-C1'-N1-C2) of the snapshots shown in Figure 4 in the main text, as well as their mean values and standard deviations over 200.5 - 1000.5 ns.

Analysis of the simulated sugar puckers of the $^{6}\underline{U}$ nucleotides of duplex **6** in the structure representing the most populated 'minor groove' cluster (37% of the snapshots of the simulation of **6** starting from A-DNA) shown in main text Figure 4a (cyan) revealed a local C3′-*endo* conformation with phase angles of 56° (C4'-*exo*) and 13° (C3'-*endo*), with mean phase angles of 33 ± 42° and 35 ± 42° over 200.5 - 1000.5 ns of the simulation. This is consistent with the X-ray data (49° and 36°, respectively). The rest of the duplex adopts B-form DNA conformations (mean phase angles 148 ± 6°, C2′-*endo*). The sugar puckers of the $^{5}\underline{U}$ nucleotides in the minor groove conformation of duplex **5** exhibit similar phase angles; in the snapshot shown in main text Figure 4c, angles of 19° and 29° are observed (mean 31 ± 42° and 32 ± 47°, 200.5 - 1000.5 ns), again with the rest of the system adopting B-form DNA conformations (147 ± 6°, C2′-*endo*). These findings mirror the pseudorotation angle observed for the $^{5}\underline{U}$ nucleotide in the crystal structure of the singly modified duplex **7** (45°).

Analysis of the glycosidic torsion angles also revealed excellent agreement between the MD simulations and the X-ray structures. For the minor groove label conformation of duplex **6** as shown in Figure 4a in the main text, torsion angles of -145° (mean -144 ± 9°) and -155° (mean -143 ± 9°) were obtained for the two $^{6}\underline{U}$ nucleotides, compared to values of -141° and -146° in the X-ray structure (Figure 3b). Angles of -139° (mean -144 ± 9°) and -144° (mean -145 ± 10°) for duplex **5** also match very well with the X-ray value of -146° in duplex **7**.

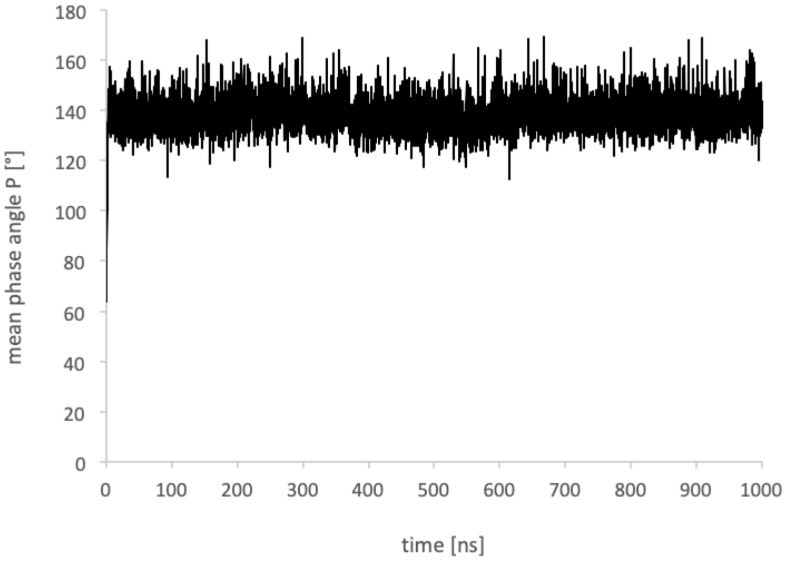

Figure S18. Mean value of the phase angles describing the sugar pucker of all residues vs. time. Simulation of system **6** with the spin labels in the minor groove (first of three independent runs).

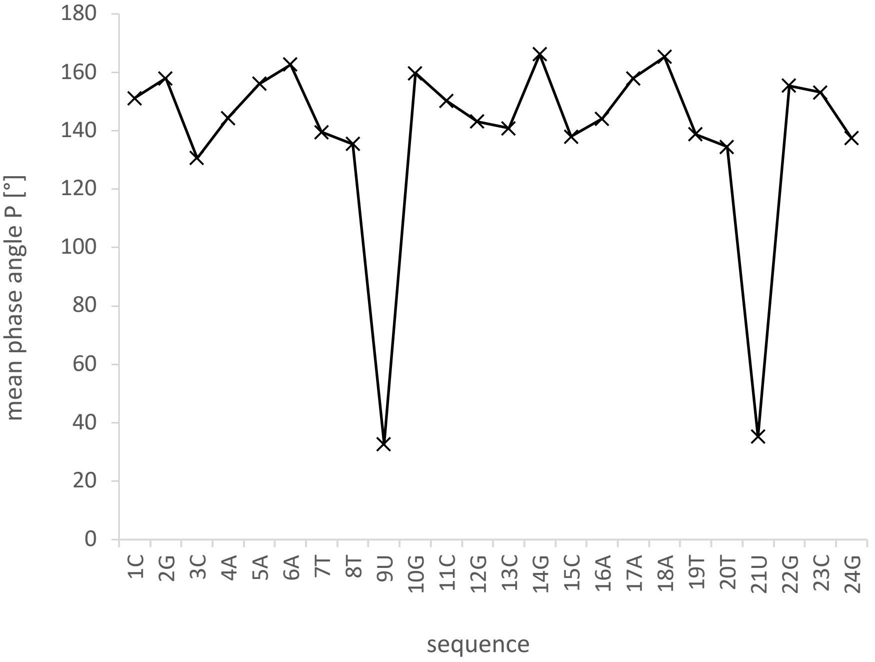

Figure S19. Phase angles of each DNA residue (mean values over 200.5-1000.5 ns) versus sequence. Simulation of duplex **6**, minor groove spin label conformation (first of three independent runs).

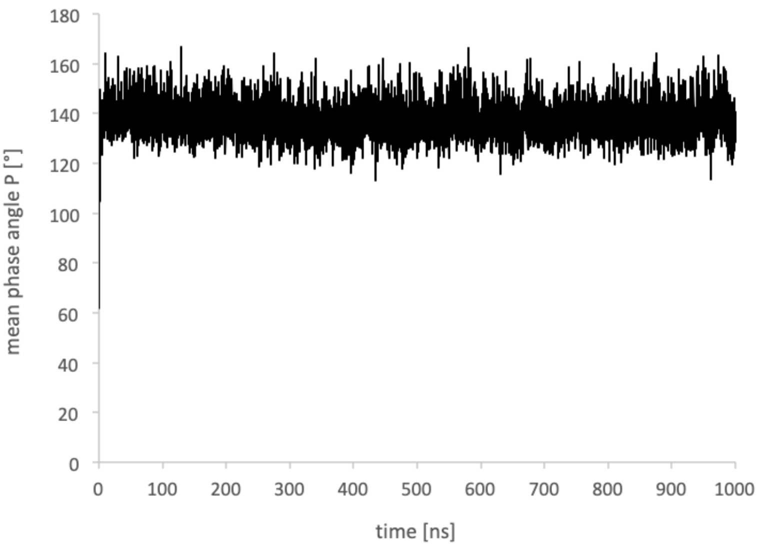

Figure S20. Mean value of the phase angles describing the sugar pucker of all residues vs. time. Simulation of system **5** with the spin labels in the minor groove (first of three independent runs).

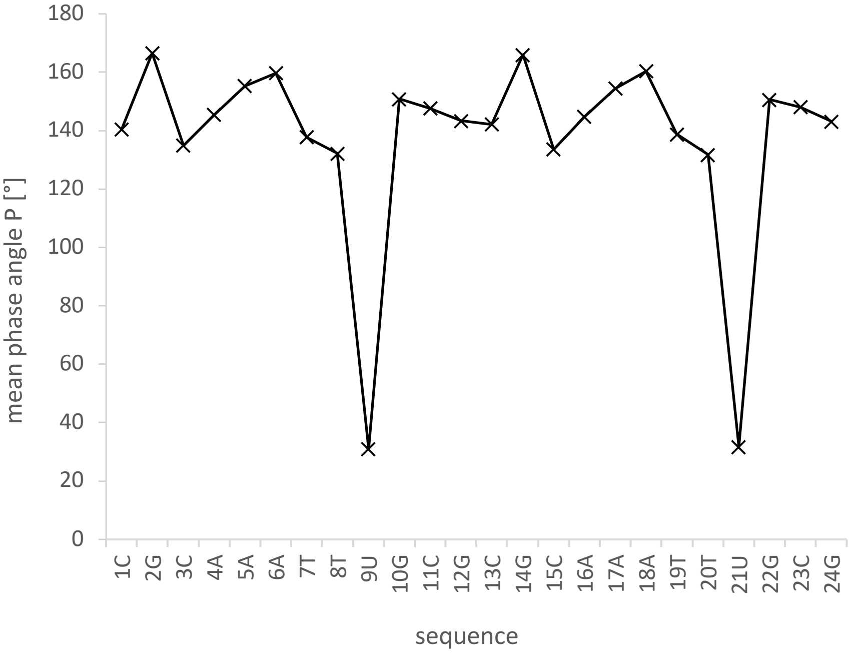

Figure S21. Phase angles of each DNA residue (mean values over 200.5-1000.5 ns) versus sequence. Simulation of duplex **5**, minor groove spin label conformation (first of three independent runs).

Table S10. Phase angles for modified nucleotides in the snapshot in Figure 4 in the main text, and mean / standard deviations over 200.5 – 1000.5 ns (first of three independent runs). [a] Phase angle in duplex **7**.

Duplex / nt	Snapshot phase angle	Sugar conformation	Mean phase angle (±1 SD)	X-ray phase angle	Sugar conformation
**5** / 9^{5}U	19	C3'-*endo*	31±42°	45[a]	C4'-*exo*
**5** / 21^{5}U	29	C3'-*endo*	32±47°		
**5** / rest of duplex	–	C2'-*endo*	147±6°	–	–
**6** / 9^{6}U	56	C4'-*exo*	33±42°	49	C4'-*exo*
**6** / 21^{6}U	13	C3'-*endo*	35±42°	36	C3'-*endo*
**6** / rest of duplex	–	C2'-*endo*	148±6°	–	–

Table S11. Glycosidic torsion angles for the modified nucleotides snapshot in Figure 4 in the main text, and mean / standard deviations over 200.5 – 1000.5 ns (first of three independent runs). [a] Glycosidic torsion angle in duplex **7**.

Duplex / nt	Glycosidic torsion angle	Mean glycosidic torsion angle (±1 SD)	X-ray torsion angle
**5** / 9^{5}U	-139°	-144±9°	-146°[a]
**5** / 21^{5}U	-144°	-145±10°	
**6** / 9^{6}U	-145°	-144±9°	-141°
**6** / 21^{6}U	-155°	-143±9°	-146°

Analysis of spin label conformations. Plots of the dihedral angles describing rotations around the single bonds between the ring systems of the spin labels are given in Figure S22 and S23 (for the first of three runs of the simulations starting from A-DNA of **6** and **5**, respectively). For **6** (Figure S22), we analyzed the rotations of the bonds between ribose C2' and the triazole ring in the spin label (C1‘-C2‘-C11-N5) and between the triazole ring and piperidine (N4-N30-C13-C14). For **5** (Figure S23) we analyzed the dihedrals describing rotations around the bond between ribose C2' and the triazole ring (C1‘-C2‘-C11-N5), between the triazole ring and the CH_2-group (N4-N30-C13-C17) and between the CH_2-group and the pyrroline ring (N30-C13-C17-C16). Visual inspection of the trajectories and of the volume visited by the NO-group of the spin labels in the simulations (Figures S13 and S15) indicates that rotations around the spin label single bonds are sufficiently sampled in the simulations. Especially the dihedrals describing rotations of the bonds between triazole and piperidine (in **6**) and between triazole and CH_2 and between CH_2 and pyrroline (in **5**) exhibit significant rotational variation and adopt at least two major conformations each. Only the bond between the sugar C2' and the triazole ring does not reach a different major conformation most of the simulation time (with the exception of $21^5\underline{U}$, where two major conformations are observed and very rare flips in $9^6\underline{U}$ and $21^6\underline{U}$), while still moving constantly. Here, the nitrogen atoms in the triazole rings remain solvent-exposed, while C-H points towards the inner part of the helix, a conformation that is possibly stabilized by solvation and hydrophobic effects. It should be noted that structural variation is always a compromise between all possible degrees of freedom, e.g. between variation in the dihedrals and nonbonding interactions. In the spatially restricted environment of the minor groove, the spin labels can only adopt conformations that do not lead to Van der Waals clashes with the DNA backbone.

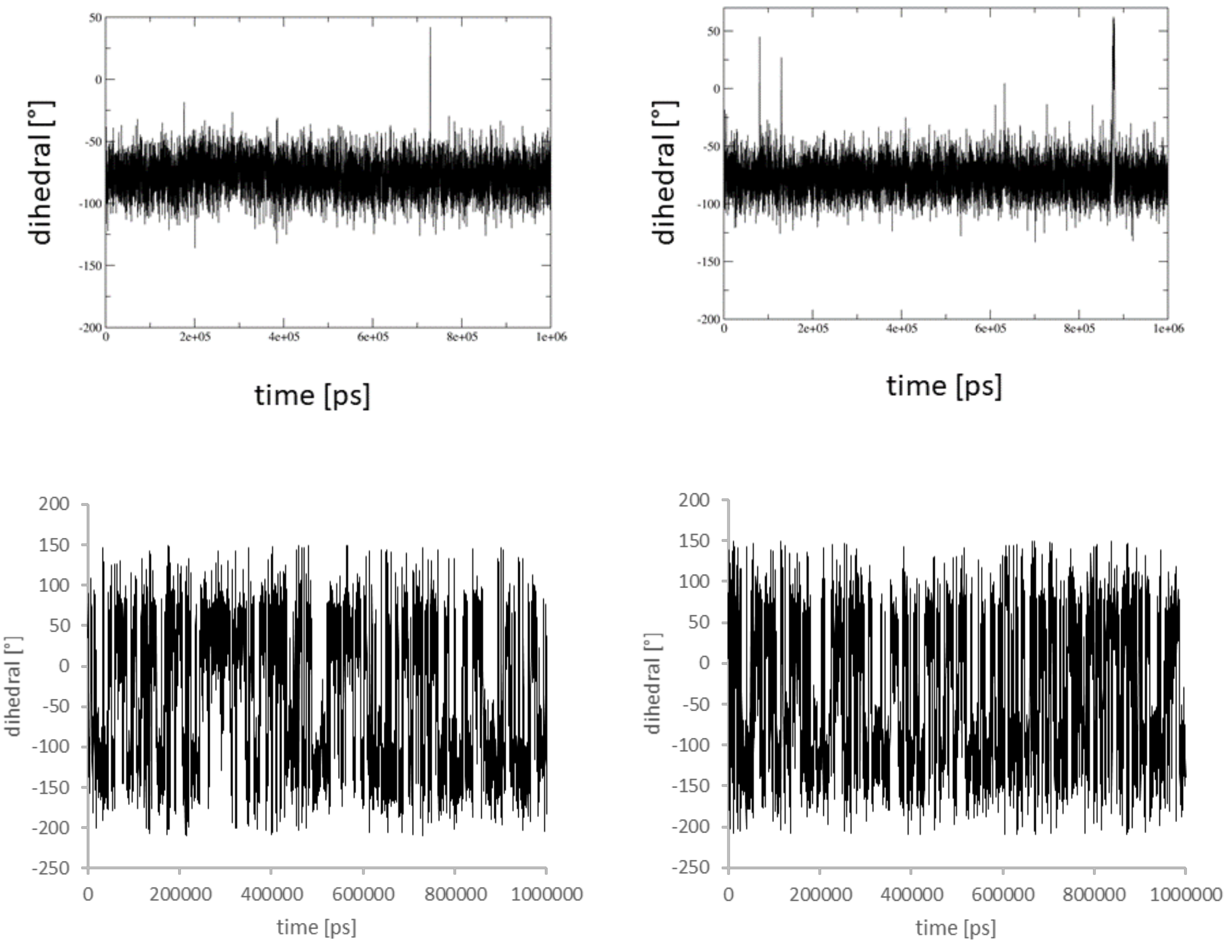

Figure S22. Dihedral C1'-C2'-C11-N5 (for definition, see text) in spin label 6-Me in 9^6U (above, left) and 21^6U (above, right) and dihedral N4-N30-C13-C14 in 9^6U (below, left) and 21^6U (below, right). Simulation of duplex **6**, minor groove spin label conformation (first of three independent runs). In plots with frequent flips between + 180° and -180°, values > 150° were replaced by their values minus 360° to improve clarity.

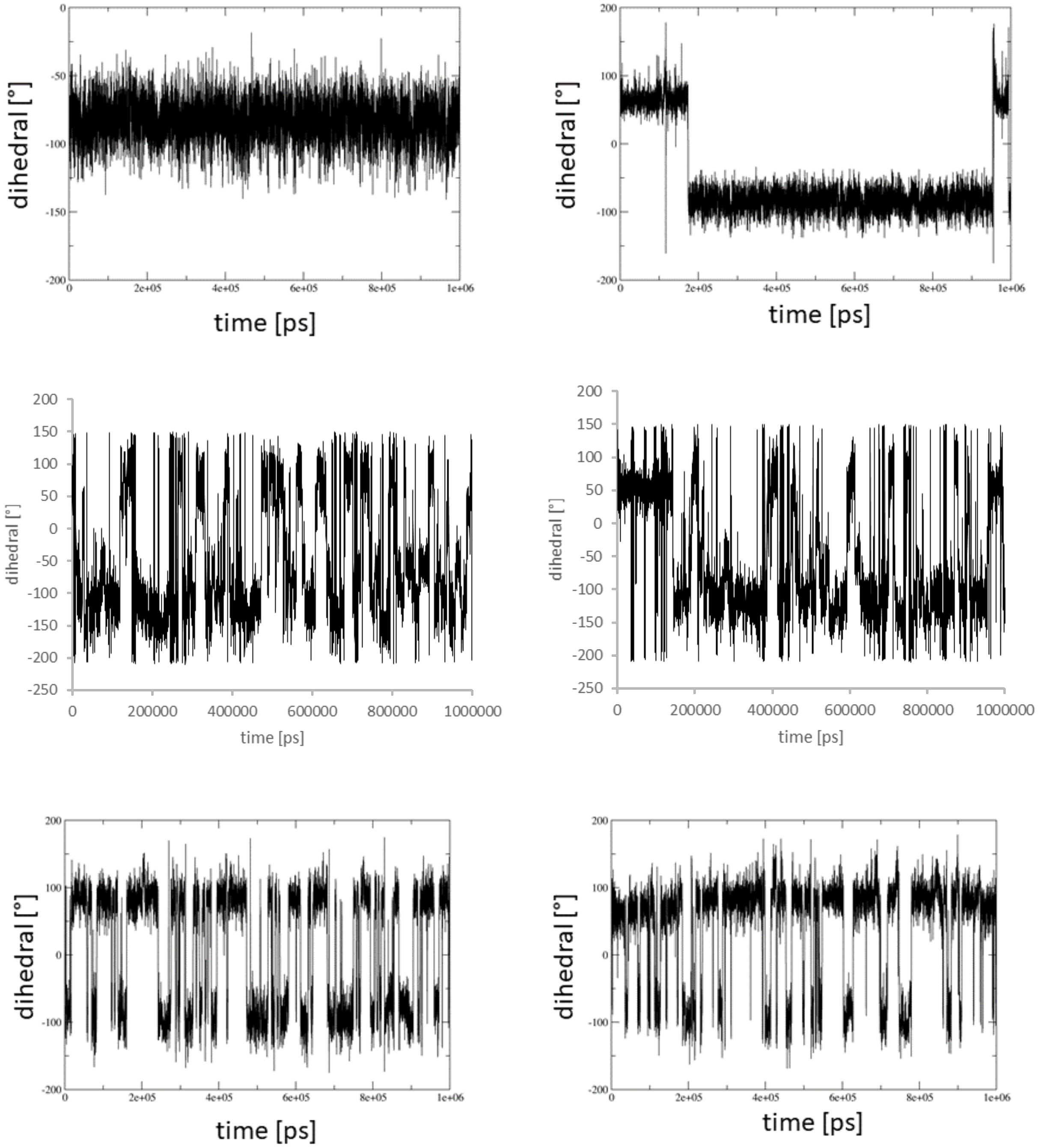

Figure S23. Dihedral C1‘-C2‘-C11-N5 (for definition, see text) in spin label 5-Me in 9^5<u>U</u> (above, left) and 21^5<u>U</u> (above, right), dihedral N4-N30-C13-C17 in 9^5<u>U</u> (middle, left) and 21^5<u>U</u> (middle, right) and dihedral N30-C13-C17-C16 in 9^5<u>U</u> (below, left) and 21^5<u>U</u> (below, right). Simulation of duplex **5**, minor groove spin label conformation (first of three independent runs). In plots with frequent flips between + 180° and -180°, values > 150° were replaced by their values minus 360° to improve clarity.

5. NMR spectroscopy

Three 14-mer oligodeoxynucleotides containing the Mbp1 binding site(56) were synthesized and purified as described above: **S1**. dCAATGACGCGTAAG; **S2**. dCT^{5NO}U̲ACGCGTCATTG; **S3**. dCT^{6NO}U̲ACGCGTCATTG. Strand **S1** is complementary to stands **S2** and **S3**, which were modified at position 3 by substitution of a 2'-spin-labelled uridine residue (denoted ^{5NO}U̲ or ^{6NO}U̲) for thymidine, as shown in Figure S24. Lyophilized oligonucleotides were dissolved in 20 mM sodium phosphate, 80 mM KCl pH 7 in 99.9% D_2O + 23 μM DSS (2,2' dimethyl silapentane-5-sulfonate), and duplexes **9** and **10** were formed by mixing equimolar amounts of oligo **S1** with oligos **S2** and **S3** respectively, followed by slow annealing from 60 °C. All NMR spectra were recorded at 14.1 T on an Agilent DD2 spectrometer using a 3 mm inverse triple resonance HCN cold probe.

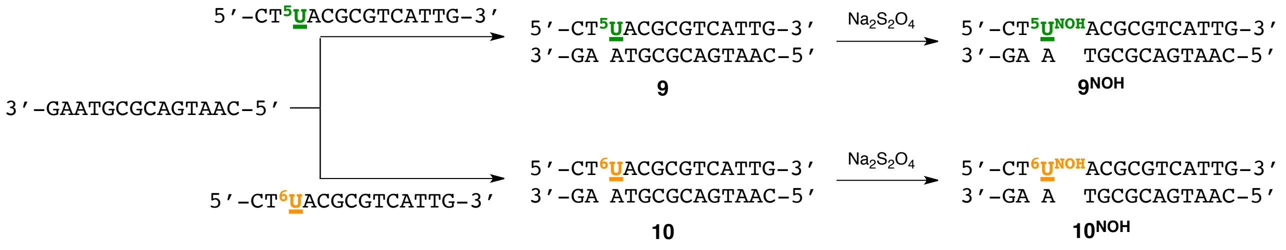

Figure S24. Synthesis of duplexes **9**,**10**, **9^{NOH}** and **10^{NOH}**.

Samples were placed in 3 mm NMR tubes for analysis. After recording spectra of the nitroxide-containing duplexes, 5 μL aliquots of 1 M sodium dithionite dissolved in the same buffer were added to reduce the nitroxide to the hydroxylamine diamagnetic state, and NMR spectra were recorded again on the reduced (hydroxylamine) form of the duplexes. 4,4-Dimethyl-4-silapentane-1-sulfonic acid (DSS) was used as an internal reference, and to assess residual magnetic field homogeneity. Samples in D_2O were recorded at 30 °C. 1D NMR spectra were recorded over a spectral width of 6 kHz with an acquisition time of 2 s and a relaxation delay of 2 s, during which residual HOD was suppressed using a weak coherent rf field.

NOESY spectra were recorded over a spectral width of 6 kHz with presaturation with acquisition times of 1 s in t_2 and 43 ms (512 increments) in t_1, and a relaxation delay of 2 s, and with mixing

times of 200 ms and 50 ms. The data were processed with 1 linear prediction in t_1, zero filling to 16kx2k complex points, with upshifted Gaussian and 1 Hz line broadening exponential.

DQCOSY spectra were recorded over a spectral width of 5.5 kHz with acquisition times of 1 s in t_2 and 46.5 ms in t_1 (512 increments), with a relaxation delay of 1 s. Data were processed with linear prediction in t_1, zero filling to 16k x 2k points and apodization with an unshifted Gaussian and 0.5 Hz line broadening exponential in both dimensions.

Spectra in 95% ^1H_2O / 5% ^2H_2O were recorded over a spectral width of 13 kHz at 10 °C. 1D spectra were recorded with an acquisition time of 2 s and a relaxation delay of 2 s, using excitation sculpting to suppress the H_2O resonance; NOESY spectra were also recorded using excitation sculpting with acquisition times of 0.5 s in t_2 and 46 ms in t_1, a relaxation delay of 1.5 s, and a mixing time of 250 ms.

Chemical shifts of the unmodified 14-mer duplex were obtained from published assignments.(56) Sugar conformations were assessed as S/N mixtures using sums of coupling constants derived from DQF-COSY, NOESY and 1D spectra, and critical distance estimates from the 50 ms NOESY mixing time experiments (especially r(1'4')) as previously described.(57,58)

NMR assignments at 30 °C

Resonances were assigned using NOESY and DQF COSY as described in the methods.

Table S12. Reduced ^{6NO}U3 duplex, $\mathbf{10^{NOH}}$.

**Nt**	**H8/6**	**H5/Me**	**H1'**	**H2'**	**H2"**	**H3'**	**H4'**	$\Sigma_{1'}$ [a]	f_s [b]
				**ppm**				**Hz**	
**C1**	7.70	5.82	5.98	2.15	2.52	4.66	4.10		
**T2**	7.56	1.76	6.03	2.44	2.52	4.94	4.27	14	0.71
^{6NOH}**U3**	7.93	5.83	6.24	3.82	-	4.86	4.65	6.5 [c]	0.5
**A4**	8.26	7.31	6.20	2.57	2.82	4.86	4.47	15.5	
**C5**	7.29	5.25	5.56	2.0	2.32	4.80	4.1	14.7	0.83
**G6**	7.82	-	5.86	2.58	2.70	4.94	4.33		
**C7**	7.26	5.30	5.67	2.0	2.36	4.81	4.11	14.9	0.86
**G8**	7.86	-	5.96	2.59	2.77	4.94			
**T9**	7.19	1.39	5.96	2.07	2.47	4.83		15.1	0.90
**C10**	7.55	5.66	5.54	2.14	2.45	4.85	4.1		
**A11**	8.32	7.57	6.25	2.71	2.94	5.02	4.42	14.5	0.8
**T12**	7.21	1.44	5.94	2.00	2.49	4.83			
**T13**	7.30	1.68	5.89	2.01	2.37	4.86			
**G14**	7.91	-	6.15	2.59	2.4	4.68	4.44	14.7	0.83
**C15**	7.65	5.94	5.64	1.83	2.36	4.69	4.04	14.7	0.83
**A16**	8.33	7.38	5.92	2.82	2.93	5.03	4.47	15.5	0.97
**A17**	8.20	7.70	6.19	2.59	2.89	5.02	4.47	15.5	0.97
**T18**	6.99	1.34	5.65	1.83	2.29	4.82	4.11	14.7	0.83
**G19**	7.80	-	5.48	2.61	2.72	4.97	4.43	14.7	0.83
**A20**	8.13	7.73	6.16	2.61	2.84	5.02	4.44		
**C21**	7.15	5.16	5.54	1.89	2.28	4.79	4.1	14.6	0.81
**G22**	7.81	-	5.85	2.60	2.71	4.94			
**C23**	7.25	5.31	5.68	1.99	2.36	4.82	4.11	15.5	0.97
**G24**	7.87	-	5.95	2.58	2.69	4.94			
**T25**	7.11	1.54	5.81	1.72	2.12			16	1
**A26**	8.07		5.87	2.55	2.62	4.91	4.33		
**A27**	8.02		5.97	2.52	2.65	4.92	4.4		
**G28**	7.74	-	5.96	2.51	2.33	4.64			

[a] $\Sigma_{1'} = {}^3J_{1'2'} + {}^3J_{1'2''}$

[b] $f_s = (\Sigma_{1'} - 9.8)/5.9$ = apparent mol fraction of the 'S' conformer for a 2-state equilibrium

[c] ${}^3J_{1'2'} = 6.5 \pm 0.5$, ${}^3J_{2'3'} = 5 \pm 1$ Hz from COSY and NOESY

All other $\Sigma_{1'} > 14.5$ Hz, $\Sigma 2' > 28$ Hz => 'S' with $f_s > 0.8$

Chemical shifts of the spin-label 2,6 Me groups: 1.13, 1.15 ppm

NOEs were observed to 3,5 protons at 1.93, 2.06 ppm

Table S13. Reduced $^{5NO}\underline{U}3$ duplex, $\mathbf{9^{NOH}}$

Resonances were assigned using NOESY and DQF COSY as described in the methods.

Nt	H8/6	H5/Me	H1'	H2'	H2"	H3'	H4'	Σ1' [a]	f_s [b]
				ppm				Hz	
**C1**	7.70	5.83	5.96	2.11	2.48	4.64			
**T2**	7.50	1.73	5.95	2.14	2.52	4.93	4.36		
**^{5NOH}U3**	7.94	5.83	6.33	3.92	-	4.87		7.5 [c]	0.5
**A4**	8.26	7.31	6.20	2.57	2.83	4.87	4.47	>14	>0.71
**C5**	7.29	5.27	5.60	2.00	2.32	4.81		15.6	0.98
**G6**	7.82	-	5.86	2.57	2.68	4.94	4.34		
**C7**	7.25	5.30	5.65	1.99	2.36	4.81	4.11	14.5	0.8
**G8**	7.85	-	5.95	2.61	2.78	4.93	4.36		
**T9**	7.19	1.39	5.97	2.08	2.47	4.84			
**C10**	7.54	5.65	5.53	2.13	2.42	4.89		15.5	0.97
**A11**	8.32	7.57	6.24	2.72	2.93	5.02	4.43	15.5	0.97
**T12**	7.21	1.42	5.93	1.98	2.47	4.84			
**T13**	7.30	1.67	5.89	2.00	2.38	4.87			
**G14**	7.90	-	6.14	2.6	2.36	4.69		15.0	0.88
**C15**	7.65	5.94	5.64	1.82	2.34	4.69		>14	>0.71
**A16**	8.32	7.38	5.91	2.81	2.93	5.03	4.38		
**A17**	8.20	7.70	6.19	2.58	2.88	5.02	4.47	>14.7	>0.83
**T18**	6.98	1.35	5.65	1.84	2.27	4.82	4.11	>14	>0.71
**G19**	7.80	-	5.48	2.61	2.73	4.98		>14	>0.71
**A20**	8.13	7.73	6.16	2.60	2.84	5.02	4.44		
**C21**	7.14	5.14	5.53	1.89	2.29	4.80	4.11	16	1
**G22**	7.81	-	5.85	2.59	2.69	4.92	4.33		
**C23**	7.25	5.28	5.69	1.98	2.36	4.81		15.5	0.97
**G24**	7.87	-	5.94	2.57	2.69	4.93	4.36		
**T25**	7.10	1.54	5.82	1.76	2.11			>15	>0.88
**A26**	8.08		5.91	2.53	2.60	4.88			
**A27**	8.03		6.01	2.51	2.63	4.93			
**G28**	7.74	-	5.96		2.35	4.63			

[a] $\Sigma_{1'} = {}^3J_{1'2'} + {}^3J_{1'2''}$

[b] $f_s = (\Sigma_{1'} - 9.8)/5.9$ = apparent mol fraction of the 'S' conformer for a 2-state equilibrium

[c] ${}^3J_{1'2'} = 7.5 \pm 0.5$ from COSY and NOESY

All Σ1'>14-15 Hz Σ2'>27-30 Hz => 'S' with f_s>0.7-0.88

Chemical shifts of the spin label 2,5 Me resonances were at 1.22, 1.30 ppm

Table S14. Assignments of exchangeable protons at 10 °C in H_2O for duplexes **10**$^{\mathbf{NOH}}$ and **9**$^{\mathbf{NOH}}$. Resonances were assigned using NOESY recorded at 10 °C with a mixing time of 200 ms.

Nt	N1/N3H duplex 10^{NOH}	N1/N3H duplex 9^{NOH}	Ade H2	CN4H
**C1-G28**	X[a]	X	-	X
**T2-A27**	X	X	nd	-
**U3-A26**	X	X	nd	-
**A4-T25**	14.03	14.03	7.32	-
**C5-G24**	12.94	12.94	-	8.29
**G6-C23**	12.87/88	12.87	-	8.28,6.45
**C7-G22**	12.88/87	12.87	-	8.28,6.68
**G8-C21**	12.84	12.81	-	8.10,6.48
**T9-A20**	13.73	13.73	7.70	-
**C10-G19**	12.39	12.39	-	8.43.6.85
**A11-T18**	13.41	13.40	7.54	-
**T12-A17**	13.72	13.73	7.73	-
**T13-A16**	X	X	nd	-
**G14-C15**	X	X	-	X

[a] X=exchanged with water. A4-T25 are largely exchanged with water. G24N1H shows considerable exchange. Thus, T2-C5 show weak H bonding.

Table S15. Conformation of the ^{NO}U3 residue in the two spin-labelled duplexes. Scalar couplings were determined from DQF COSY and NOESY (t_m=200 ms), distances (d) estimated from NOESY at 50 ms.

**Parameter**	**Dimension**	**Value**			
		^{6NO}U3	^{5NO}U3	"B" P=162°	"A" P=9°
$^3J_{1'2'}$	Hz	6.5±0.5	7.0±1	10	1
$^3J_{2'3'}$	Hz	6.0±1	6.0±1	6	5.5
d(H1'H4')	nm	0.32±0.02	0.33±0.02	0.32	0.34
d(H1'H2')	nm	0.29±0.02	0.3±0.02	0.30	0.29
d(H6'H2')	nm	0.25±0.01	0.25±0.01	0.21	0.36
d(H6'H3')	nm	0.33±0.02	0.31±0.02	0.41	0.31

The values of $d_{1'4'}$, $d_{1'2'}$ and the coupling constants are not consistent with a pure state with P near 90°, nor of P>198°. The data are more consistent with a conformational mixture of N and S. For ^{6NO}U3, f_s=0.6 with Ps=162, P_N=9, the expected values of $^3J_{2'3'}$ $^3J_{3'4'}$ $d_{1'4'}$ $d_{1'2'}$ would be 5.8 Hz, 4.2 Hz, 3.3 Å and 3 Å respectively. Glycosyl torsion angle T3 in the unmodified duplex ≈ -121 °, and in U3 - 140 to -160°.

Table S16. $^{6NO}\underline{U}$3 duplex **10**

Resonances were assigned using NOESY and DQF COSY as described in the methods.

Nt	H8/6	H5/Me	H1'	H2'	H2"	H3'	H4'	Σ1' [a]	f_s [b]
				ppm				Hz	
[c]									
**C7**	7.25	5.30	5.65	1.98	2.34			>15	>0.88
**G8**	7.85	-	5.96	2.60	278	4.95			
**T9**	7.19	1.39	5.96	2.08	2.48	4.84		15.5	0.97
**C10**	7.54	5.67	5.55	2.14	2.44	4.85	4.14	15.2	0.91
**A11**	8.32		6.24	2.72	2.93	5.03	4.43	14.7	0.83
**T12**	7.22	1.44	5.95	1.99	2.44	4.83	4.37		
**T13**	7.31	1.68	5.93	2.02	2.39	4.85	4.37		
**G14**	7.9		6.15	2.57	2.40	4.68		15.5	0.97
**C15**	7.64	5.93	5.65	1.81	2.33	4.68		14.5	0.8
**A16**	8.32		5.90	2.81	2.94	5.03	4.47		
**A17**	8.21		6.19	2.59	2.89	5.02	4.47		
**T18**	6.99	1.35	5.66	1.85	2.28	4.82	4.14		
**G19**	7.80		5.49	2.61	2.72	4.97	4.32	14.5	0.8
**A20**	8.13		6.16	2.62	2.85	5.01	4.47	16	1
**C21**	7.15	5.15	5.54	1.90	2.29	4.79	4.16	15.1	0.9
**G22**	7.81		5.86	2.59	2.70	4.95	4.33	>15	>0.88
**C23**	7.25	5.31	5.65	1.99	2.36	4.82			
**G24**			5.95	2.6	2.78	4.94	4.37		

[a] $\Sigma_{1'} = {}^3J_{1'2'} + {}^3J_{1'2''}$

[b] $f_s = (\Sigma_{1'} - 9.8)/5.9$ = apparent mol fraction of the ‘S’ conformer for a 2-state equilibrium

[c] Resonances from G1-G6 and T25-G28 were broadened beyond detection.

Table S17. ^{5NO}U3 duplex **9.**

Resonances were assigned using NOESY and DQF COSY as described in the methods.

Nt	H8/6	H5/Me	H1'	H2'	H2"	H3'	H4'	Σ1' [a] Hz	f_s [b]
				ppm					
[c]									
**G6**						4.96			
**C7**	7.25	5.30	5.65	1.99	2.34	4.84	4.11	15	0.88
**G8**	7.85	-	5.96	2.61	2.78	4.95	4.37		
**T9**	7.19	1.39	5.66	2.07	2.37	4.85	4.11		
**C10**	7.54	5.66	5.55	2.13	2.44	4.85	4.14	14.7	0.83
**A11**	8.32		6.22	2.72	2.93	5.03	4.42	15.4	0.95
**T12**	7.21	1.43	5.95	1.98	2.47	4.83			
**T13**	7.31	1.68	5.93	2.00	2.39	4.85	4.38		
**G14**	7.91		6.15	2.60	2.37				
**C15**	7.63	5.92	5.65	1.81	2.33	4.67		15	0.88
**A16**	8.32		5.91	2.81	2.94	5.02		15	0.88
**A17**	8.21		6.19	2.58	2.90	5.02	4.47	15.1	0.9
**T18**	6.99	1.35	5.66	1.85	2.29	4.83	4.11		
**G19**	7.80		5.49	2.61	2.72	4.97	4.32	15.1	0.9
**A20**	8.13		6.16	2.62	2.85	5.01	4.43	15.0	0.88
**C21**	7.15	5.15	5.54	1.89	2.29	4.79	4.33	15.1	0.9
**G22**	7.81		5.86	2.59	2.70	4.95		15.5	0.97
**C23**	7.25	5.30	5.65	1.99	2.35	4.82	4.11	15.3	0.93

[a] $\Sigma_{1'} = {}^3J_{1'2'} + {}^3J_{1'2''}$

[b] $f_s = (\Sigma_{1'} - 9.8)/5.9$ = apparent mol fraction of the 'S' conformer for a 2-state equilibrium

[c] Resonances from C1-G6 and G25-G28 were broadened beyond detection

Chemical shift differences between the two different spin-labelled duplexes in the diamagnetic and paramagnetic states. Data were taken from Tables S12, S13, S16, S17, and Chernatynskaya, A.V. (2009).(56) The chemical shift perturbation at ^{NO}U3 due to the differences in the 2' spin label are marginal, implying very similar average conformations.

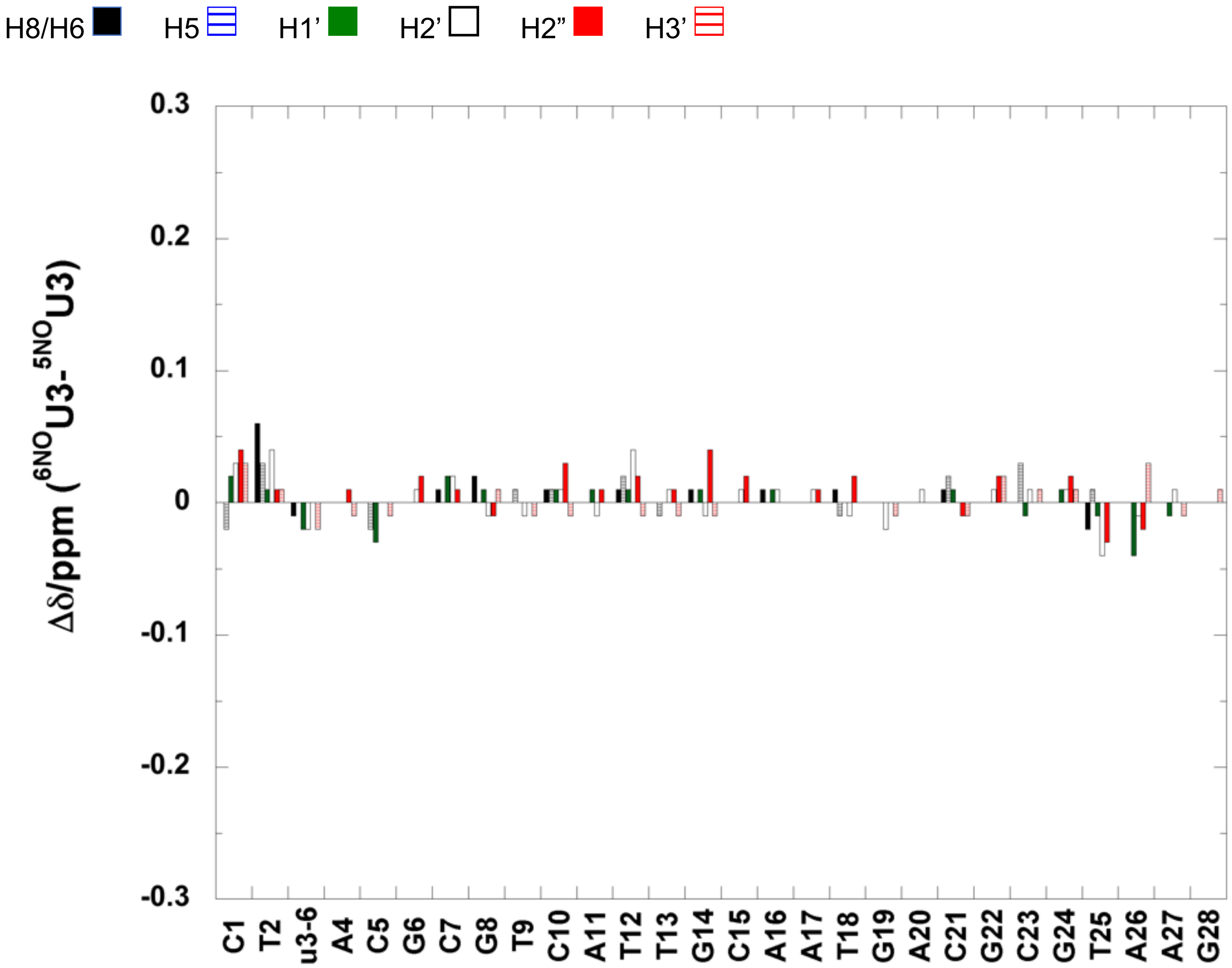

Figure S25. Chemical shift difference for **10^{NOH} – 9^{NOH}**.

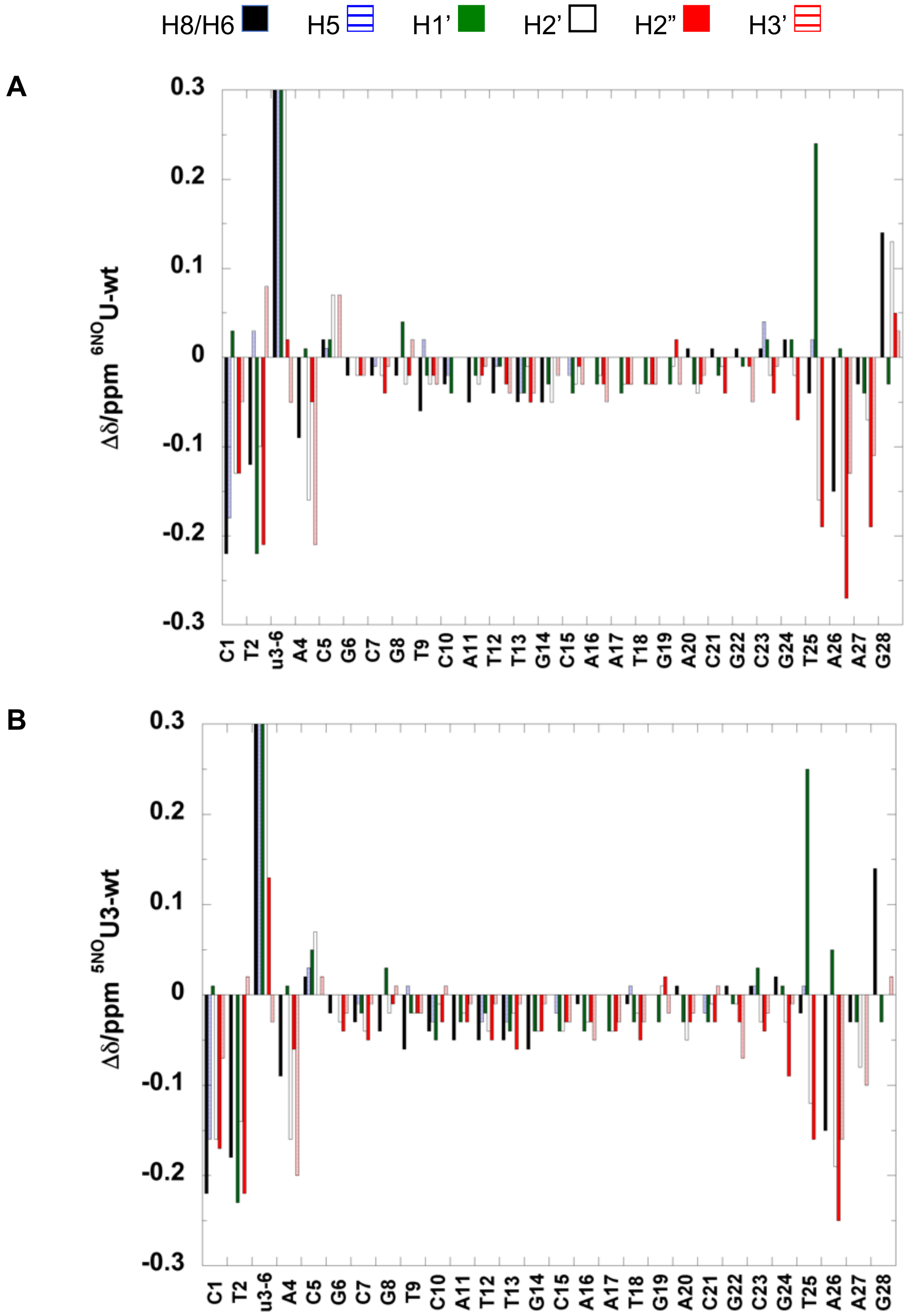

Figure S26. Chemical shift difference for **10^{NOH}–11** and **9^{NOH}–11** (Figures A and B, respectively). Data taken from Tables S12 and S13, and Chernatynskaya, A.V. (2009).(56) Chemical shift differences are significant only at U3 and its nearest neighbors (on both strands), implying that the altered conformation of U3 compared with T3 does not propagate beyond the nearest neighbour.

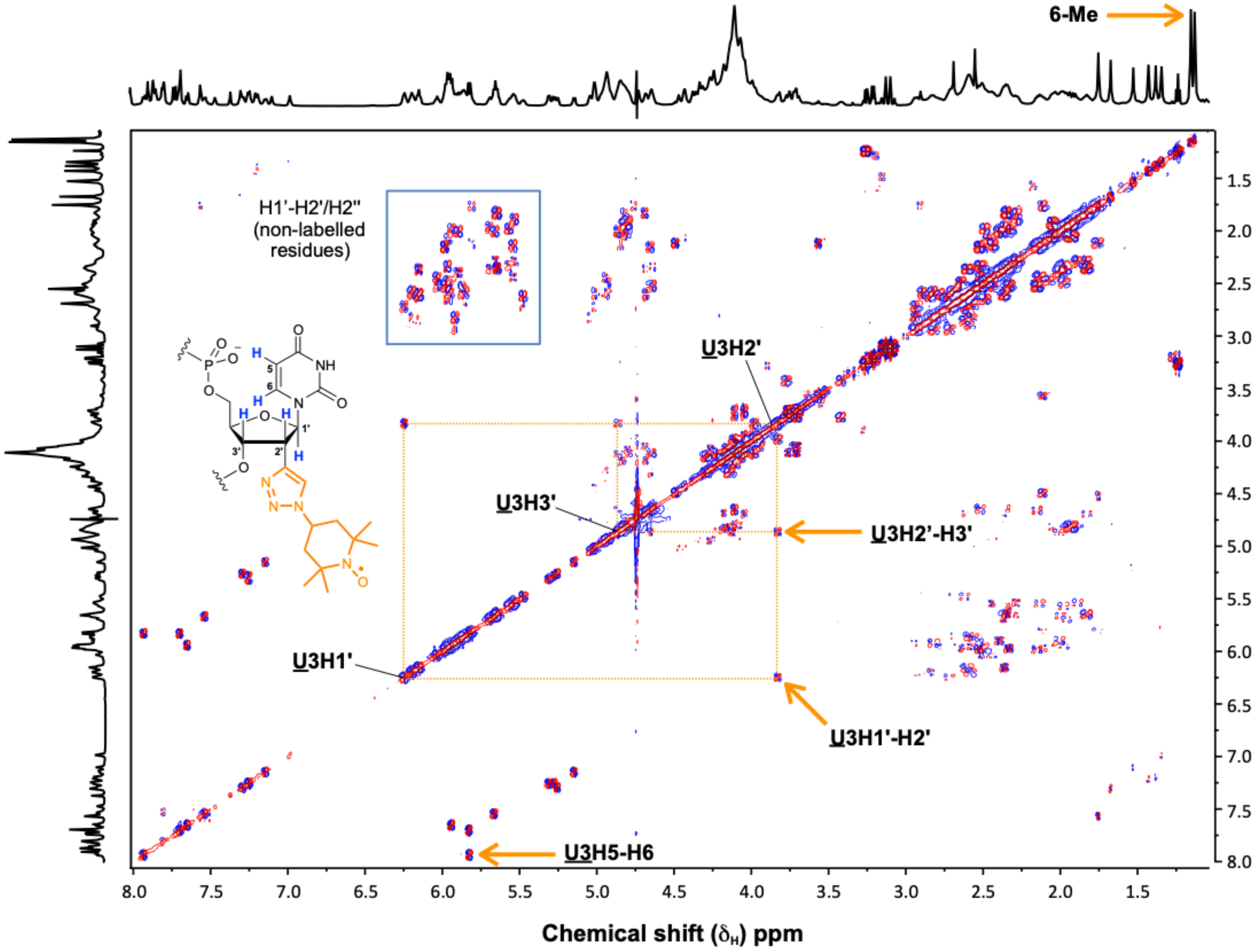

Figure S27. Double Quantum filtered COSY of the reduced 6Me duplex **10**^{NOH}, recorded at 30 °C, 14.1 T with an acquisition time of 1 s in t_2, as described in the Methods. 6Me are the methyl resonances of the reduced TEMPO unit of the modified nt. Cross peaks between H1' H2'/H2'' H2'-H3' and H3'-H4'; are consistent with S-type sugar puckers for all nucleotides except U3.

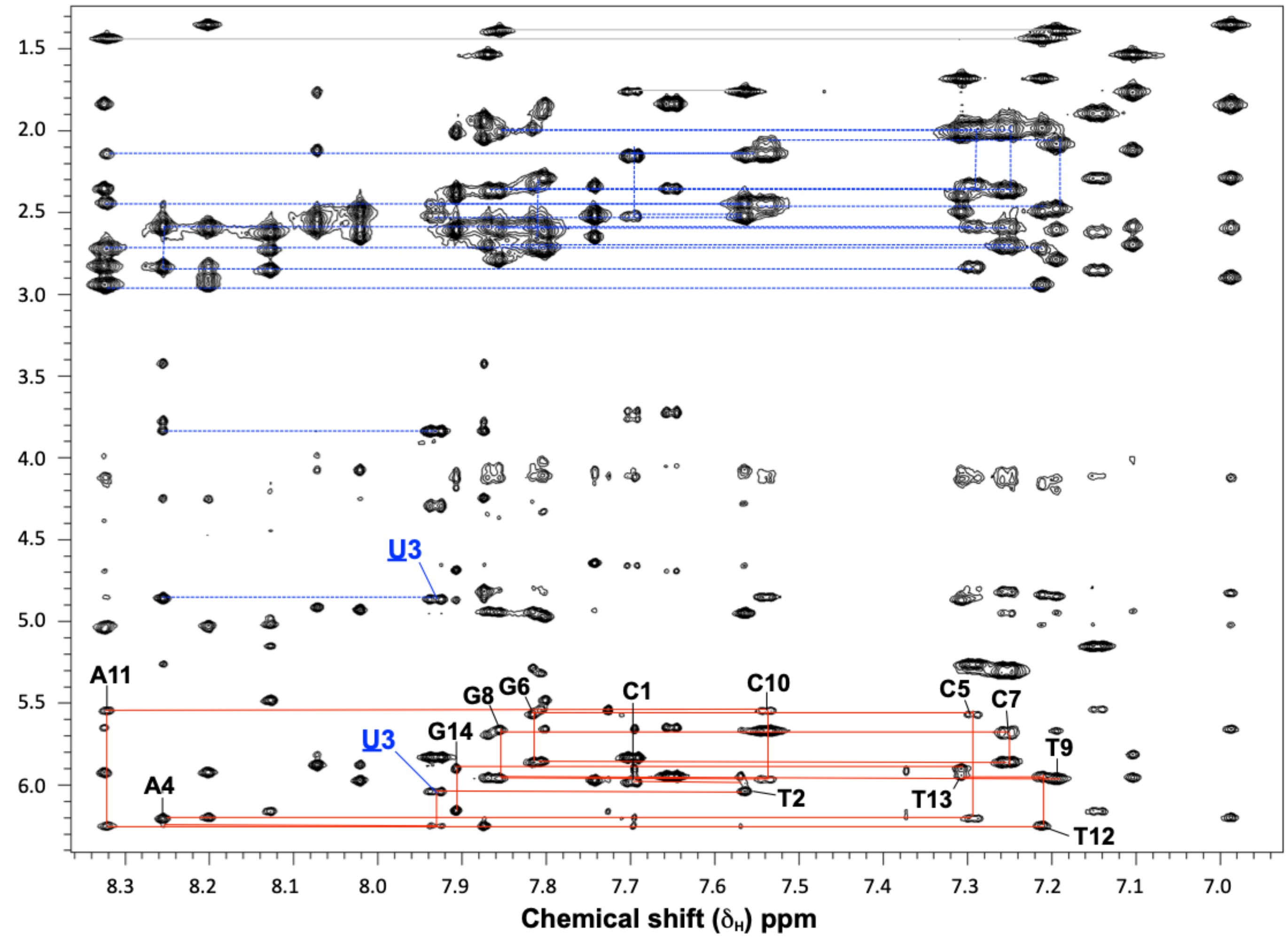

Figure S28 ^{1}H–^{1}H NOESY spectrum of duplex **10^{NOH}**, recorded at 30 °C, 14.1 T with an acquisition time of 1 s in t_2, and a mixing time of 200 ms, as described in the Methods. Assignments of the modified strand are shown. Dashed lines show sequential H2'/H2" to H8/6 connectivities. The relative cross peak intensities are typical of a B-like duplex, with the exception of the ^{6NO}U3 residue which shows non B-like properties as described in the text

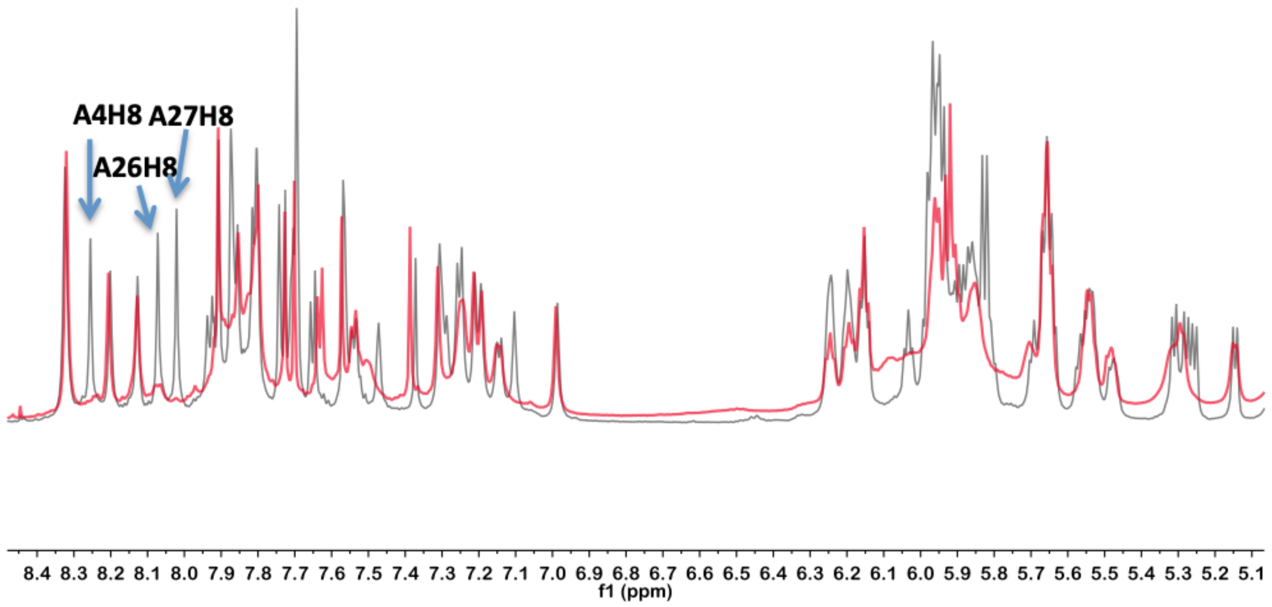

Figure S29. Overlay of ^{1}H NMR spectra of duplex **10** in the oxidized (**10,** red) and reduced (**10**$^{\mathbf{NOH}}$, grey) states at 30 °C, 14.1 T as described in the text. The oxidized form (nitroxide) has fewer peaks than the reduced form (hydroxylamine) owing to paramagnetic broadening.

Data availability

The structure factors and coordinates of the tetramethylpiperidinoxyl and tetramethylpyrrolinoxyl labelled DNA duplexes have been deposited in the Protein Data Bank under accession codes 6QJS and 6QJR. NMR data have been deposited with the Biological Magnetic Resonance Data Bank under accession number 27958. All other data are available upon request from the corresponding authors.

6. References

1. Haugland,M.M., El-Sagheer,A.H., Porter,R.J., Peña,J., Brown,T., Anderson,E.A. and Lovett,J.E. (2016) 2′-Alkynylnucleotides: A sequence- and spin label-flexible strategy for EPR spectroscopy in DNA. *J. Am. Chem. Soc.*, **138**, 9069-9072.
2. Haq,I., Ladbury,J.E., Chowdhry,B.Z., Jenkins,T.C. and Chaires,J.B. (1997) Specific binding of hoechst 33258 to the d(CGCAAATTTGCG)$_2$ duplex: calorimetric and spectroscopic studies. *J. Mol. Biol.*, **271**, 244-257.
3. Fidanza,J.A. and McLaughlin,L.W. (1989) Introduction of reporter groups at specific sites in DNA containing phosphorothioate diesters. *J. Am. Chem. Soc.*, **111**, 9117-9119.
4. Milov,A.D., Salikhov,K.M. and Shirov,M.D. (1981) Application of ELDOR in electron-spin echo for paramagnetic center space distribution in solids. *Fiz. Tverd. Tela*, **23**, 975-982.
5. Martin,R.E., Pannier,M., Diederich,F., Gramlich,V., Hubrich,M. and Spiess,H.W. (1998) Determination of end-to-end distances in a series of TEMPO diradicals of up to 2.8 nm length with a new four-pulse double electron electron resonance experiment. *Angew. Chem. Int. Ed.*, **37**, 2833-2837.
6. Jeschke,G., Chechik,V., Ionita,P., Godt,A., Zimmermann,H., Banham,J., Timmel,C.R., Hilger,D. and Jung,H. (2006) DeerAnalysis2006—a comprehensive software package for analyzing pulsed ELDOR data. *Appl. Magn. Reson.*, **30**, 473-498.
7. Hilger,D., Jung,H., Padan,E., Wegener,C., Vogel,K.-P., Steinhoff,H.-J. and Jeschke,G. (2005) Assessing oligomerization of membrane proteins by four-pulse DEER: pH-dependent dimerization of NhaA Na^+/H^+ antiporter of E. coli. *Biophys. J.*, **89**, 1328-1338.
8. Kabsch,W. (2010) Integration, scaling, space-group assignment and post-refinement. *Acta Crystallogr., Sect. D: Biol. Crystallogr.*, **66**, 133-144.
9. Evans,P.R. and Murshudov,G.N. (2013) How good are my data and what is the resolution? *Acta Crystallogr., Sect. D: Biol. Crystallogr.*, **69**, 1204-1214.
10. McCoy,A.J., Grosse-Kunstleve,R.W., Adams,P.D., Winn,M.D., Storoni,L.C. and Read,R.J. (2007) Phaser crystallographic software. *J. Appl. Crystallogr.*, **40**, 658-674.
11. Murshudov,G.N., Skubák,P., Lebedev,A.A., Pannu,N.S., Steiner,R.A., Nicholls,R.A., Winn,M.D., Long,F. and Vagin,A.A. (2011) REFMAC5 for the refinement of macromolecular crystal structures. *Acta Crystallogr., Sect. D: Biol. Crystallogr.*, **67**, 355-367.
12. Emsley,P., Lohkamp,B., Scott,W.G. and Cowtan,K. (2010) Features and development of Coot. *Acta Crystallogr., Sect. D: Biol. Crystallogr.*, **66**, 486-501.
13. Lu,X.J. and Olson,W.K. (2003) 3DNA: a software package for the analysis, rebuilding and visualization of three-dimensional nucleic acid structures. *Nucleic Acids Res.*, **31**, 5108-5121.
14. Lavery,R., Moakher,M., Maddocks,J.H., Petkeviciute,D. and Zakrzewska,K. (2009) Conformational analysis of nucleic acids revisited: Curves+. *Nucleic Acids Res.*, **37**, 5917-5929.
15. Zheng,G., Lu,X.-J. and Olson,W.K. (2009) Web 3DNA—a web server for the analysis, reconstruction, and visualization of three-dimensional nucleic-acid structures. *Nucleic Acids Res.*, **37**, W240-W246.
16. Pettersen,E.F., Goddard,T.D., Huang,C.C., Couch,G.S., Greenblatt,D.M., Meng,E.C. and Ferrin,T.E. (2004) UCSF Chimera—A visualization system for exploratory research and analysis. *J. Comput. Chem.*, **25**, 1605-1612.
17. Diederichs,K. and Karplus,P.A. (1997) Improved R-factors for diffraction data analysis in macromolecular crystallography. *Nat. Struct. Biol.*, **4**, 269-275.

18. Case,D.A., Betz,R.M., Cerutti,D.S., Cheatham,III,T.E., Darden,T.A., Duke,R.E., Giese,T.J., Gohlke,H., Goetz,A.W., Homeyer,N. *et al.* (2016) AMBER 2016. University of California, San Francisco.
19. Case,D.A., Ben-Shalom,I.Y., Brozell,S.R., Cerutti,D.S., Cheatham,III,T.E., Cruzeiro,V.W.D., Darden,T.A., Duke,R.E., Ghoreishi,D., Gilson,M.K. *et al.* (2018) AMBER 2018, University of California, San Francisco.
20. Beierlein,F.R., Paradas Palomo,M., Sharapa,D.I., Zozulia,O., Mokhir,A. and Clark,T. (2016) DNA-dye-conjugates: Conformations and spectra of fluorescence probes. *PLoS ONE*, **11**, e0160229.
21. Zozulia,O., Bachmann,T., Deussner-Helfmann,N.S., Beierlein,F., Heilemann,M. and Mokhir,A. (2019) Red light-triggered nucleic acid-templated reaction based on cyclic oligonucleotide substrates. *Chem. Commun.*, **55**, 10713-10716.
22. Cheatham,III,T.E. and Kollman,P.A. (1996) Observation of the A-DNA to B-DNA transition during unrestrained molecular dynamics in aqueous solution. *J. Mol. Biol.*, **259**, 434-444.
23. Cornell,W.D., Cieplak,P., Bayly,C.I., Gould,I.R., Merz,Jr.,K.M., Ferguson,D.M., Spellmeyer,D.C., Fox,T., Caldwell,J.W. and Kollman,P.A. (1995) A second generation force field for the simulation of proteins and nucleic acids. *J. Am. Chem. Soc.*, **117**, 5179-5197.
24. Wang,J., Cieplak,P. and Kollman,P.A. (2000) How well does a restrained electrostatic potential (RESP) model perform in calculating conformational energies of organic and biological molecules? *J. Comput. Chem.*, **21**, 1049-1074.
25. Pérez,A., Marchán,I., Svozil,D., Sponer,J., Cheatham,III,T.E., Laughton,C.A. and Orozco,M. (2007) Refinement of the AMBER force field for nucleic acids: Improving the description of α/γ conformers. *Biophys. J.,* **92**, 3817-3829.
26. Zgarbová,M., Luque,F.J., Šponer,J., Cheatham,III,T.E., Otyepka,M. and Jurečka,P. (2013) Toward improved description of DNA backbone: Revisiting epsilon and zeta torsion force field parameters. *J. Chem. Theory Comput.*, **9**, 2339-2354.
27. Krepl,M., Zgarbová,M., Stadlbauer,P., Otyepka,M., Banáš,P., Koča,J., Cheatham,III,T.E., Jurečka,P. and Šponer,J. (2012) Reference simulations of noncanonical nucleic acids with different χ variants of the AMBER force field: Quadruplex DNA, quadruplex RNA, and Z-DNA. *J. Chem. Theory Comput.*, **8**, 2506-2520.
28. Zgarbová,M., Šponer,J., Otyepka,M., Cheatham,III,T.E., Galindo-Murillo,R. and Jurečka,P. (2015) Refinement of the sugar–phosphate backbone torsion beta for AMBER force fields improves the description of Z- and B-DNA. *J. Chem. Theory Comput.*, **11**, 5723-5736.
29. Zgarbová,M., Otyepka,M., Šponer,J., Mládek,A., Banáš,P., Cheatham,III,T.E. and Jurečka,P. (2011) Refinement of the Cornell et al. nucleic acids force field based on reference quantum chemical calculations of glycosidic torsion profiles. *J. Chem. Theory Comput.*, **7**, 2886-2902.
30. Wang,J., Wolf,R.M., Caldwell,J.W., Kollman,P.A. and Case,D.A. (2004) Development and testing of a general Amber force field. *J. Comput. Chem.*, **25**, 1157-1174.
31. Wang,J., Wang,W., Kollman,P.A. and Case,D.A. (2006) Automatic atom type and bond type perception in molecular mechanical calculations. *J. Mol. Graphics Modell.*, **25**, 247-260.
32. Cieplak,P., Cornell,W.D., Bayly,C. and Kollman,P.A. (1995) Application of the multimolecule and multiconformational RESP methodology to biopolymers: Charge derivation for DNA, RNA, and proteins. *J. Comput. Chem.*, **16**, 1357-1377.
33. Bayly,C.I., Cieplak,P., Cornell,W. and Kollman,P.A. (1993) A well-behaved electrostatic potential based method using charge restraints for deriving atomic charges: the RESP model. *J. Phys. Chem.*, **97**, 10269-10280.

34. Ditchfield,R., Hehre,W.J. and Pople,J.A. (1971) Self-consistent molecular-orbital methods. IX. An extended Gaussian-type basis for molecular-orbital studies of organic molecules. *J. Chem. Phys.*, **54**, 724-728.
35. Hehre,W.J., Ditchfield,R. and Pople,J.A. (1972) Self-consistent molecular orbital methods. XII. Further extensions of Gaussian-type basis sets for use in molecular orbital studies of organic molecules. *J. Chem. Phys.*, **56**, 2257-2261.
36. Hariharan,P.C. and Pople,J.A. (1973) Influence of polarization functions on molecular-orbital hydrogenation energies. *Theor. Chim. Acta*, **28**, 213-222.
37. Hariharan,P.C. and Pople,J.A. (1974) Accuracy of AH_n equilibrium geometries by single determinant molecular-orbital theory. *Mol. Phys.*, **27**, 209-214.
38. Gordon,M.S. (1980) The isomers of silacyclopropane. *Chem. Phys. Lett.*, **76**, 163-168.
39. Francl,M.M., Pietro,W.J., Hehre,W.J., Binkley,J.S., Gordon,M.S., DeFrees,D.J. and Pople,J.A. (1982) Self-consistent molecular orbital methods. XXIII. A polarization-type basis set for second-row elements. *J. Chem. Phys.*, **77**, 3654-3665.
40. Binning,Jr.,R.C. and Curtiss,L.A. (1990) Compact contracted basis sets for third-row atoms: GA-KR. *J. Comput. Chem.*, **11**, 1206-1216.
41. Blaudeau,J.-P., McGrath,M.P., Curtiss,L.A. and Radom, L.(1997) Extension of Gaussian-2 (G2) theory to molecules containing third-row atoms K and Ca. *J. Chem. Phys.*, **107**, 5016-5021.
42. Rassolov,V.A., Pople,J.A., Ratner,M.A. and Windus,T.L. (1998) 6-31G* basis set for atoms K through Zn. *J. Chem. Phys.*, **109**, 1223-1229.
43. Rassolov,V.A., Ratner,M.A., Pople,J.A., Redfern,P.C. and Curtiss,L.A. (2001) 6-31G* basis set for third-row atoms. *J. Comput. Chem.*, **22**, 976-984.
44. Becke,A.D. (1993) Density-functional thermochemistry. III. The role of exact exchange. *J. Chem. Phys.*, **98**, 5648-5652.
45. Frisch,M.J., Trucks,G.W., Schlegel,H.B., Scuseria,G.E., Robb,M.A., Cheeseman,J.R., Scalmani,G., Barone,V., Peterson,G.A., Nakatsuji,H. *et al.* (2009). Gaussian 09. Gaussian, Inc., Wallingford, CT, USA.
46. Case,D.A., Babin,V., Berryman,J.T., Betz,R.M., Cai,Q., Cerutti,D.S., Cheatham,III,T.E., Darden,T.A., Duke,R.E., Gohlke,H. *et al.* (2014). AMBER 14. University of California, San Francisco.
47. Berendsen,H.J.C., Grigera,J.R. and Straatsma,T.P. (1987) The missing term in effective pair potentials. *J. Phys. Chem.*, **91**, 6269-6271.
48. Joung,I.S. and Cheatham,III,T.E. (2008) Determination of alkali and halide monovalent ion parameters for use in explicitly solvated biomolecular simulations. *J. Phys. Chem. B*, **112**, 9020-9041.
49. Ryckaert,J.-P., Ciccotti,G. and Berendsen,H.J.C. (1977) Numerical integration of the cartesian equations of motion of a system with constraints: molecular dynamics of n-alkanes. *J. Comput. Phys.*, **23**, 327-341.
50. Darden,T., York,D. and Pedersen,L. (1993) Particle mesh Ewald: An N·log(N) method for Ewald sums in large systems. *J. Chem. Phys.*, **98**, 10089-10092.
51. Essmann,U., Perera,L., Berkowitz,M.L., Darden,T., Lee,H. and Pedersen,L.G. (1995) A smooth particle mesh Ewald method. *J. Chem. Phys.*, **103**, 8577-8593.
52. Roe,D.R. and Cheatham,III,T.E. (2013) PTRAJ and CPPTRAJ: Software for processing and analysis of molecular dynamics trajectory data. *J. Chem. Theory Comput.*, **9**, 3084-3095.
53. Humphrey,W., Dalke,A. and Schulten,K. (1996) VMD - Visual Molecular Dynamics. *J. Mol. Graphics*, **14**, 33-38.
54. (2015) The PyMOL Molecular Graphics System, Version 1.8. Schrödinger, LLC.

55. Altona,C. and Sundaralingam,M. (1972) Conformational analysis of the sugar ring in nucleosides and nucleotides. New description using the concept of pseudorotation. *J. Am. Chem. Soc.*, **94**, 8205-8212.
56. Chernatynskaya,A.V., Deleeuw,L., Trent,J.O., Brown,T. and Lane,A.N. (2009) Structural analysis of the DNA target site and its interaction with Mbp1. *Org. Biomol. Chem.*, **7**, 4981-4991.
57. Conte,M.R., Bauer,C.J. and Lane,A.N. (1996) Determination of sugar conformations by NMR in larger DNA duplexes using both dipolar and scalar data: Application to $d(CATGTGACGTCACATG)_2$. *J. Biomol. NMR*, **7**, 190-206.
58. Hardwick,J., Lane,A.N. and Brown,T. (2018) Epigenetic modifications of cytosine: Biophysical properties, regulation and function in mammalian DNA *BioEssays*, **40**, 1700199.
